# Zigzag‐Elongated Fused π‐Electronic Core: A Molecular Design Strategy to Maximize Charge‐Carrier Mobility

**DOI:** 10.1002/advs.201700317

**Published:** 2017-11-15

**Authors:** Akito Yamamoto, Yoshinori Murata, Chikahiko Mitsui, Hiroyuki Ishii, Masakazu Yamagishi, Masafumi Yano, Hiroyasu Sato, Akihito Yamano, Jun Takeya, Toshihiro Okamoto

**Affiliations:** ^1^ Department of Advanced Materials Science Graduate School of Frontier Sciences The University of Tokyo 5‐1‐5 Kashiwanoha Kashiwa Chiba 277‐8561 Japan; ^2^ Chemistry, Materials and Bioengineering Major Graduate School of Science and Engineering Kansai University 3‐3‐35 Yamate‐cho Suita Osaka 564‐8680 Japan; ^3^ Division of Applied Physics Faculty of Pure and Applied Sciences University of Tsukuba 1‐1‐1 Tennodai Tsukuba Ibaraki 305‐8573 Japan; ^4^ National Institute of Technology Toyama College 13 Hongo‐machi Toyama Toyama 939‐8630 Japan; ^5^ Rigaku Corporation 3‐9‐12 Matsubara‐cho Akishima Tokyo 196‐8666 Japan; ^6^ PRESTO Japan Science and Technology Agency (JST) 4‐1‐8 Honcho Kawaguchi Saitama 332‐0012 Japan

**Keywords:** high carrier mobility, highly stabilized crystal phase, molecular orbital configuration, organic semiconductor, zigzag‐shaped π‐electronic core

## Abstract

Printed and flexible electronics requires solution‐processable organic semiconductors with a carrier mobility (μ) of ≈10 cm^2^ V^−1^ s^−1^ as well as high chemical and thermal durability. In this study, chryseno[2,1‐*b*:8,7‐*b*′]dithiophene (ChDT) and its derivatives, which have a zigzag‐elongated fused π‐electronic core (π‐core) and a peculiar highest occupied molecular orbital (HOMO) configuration, are reported as materials with conceptually new semiconducting π‐cores. ChDT and its derivatives are prepared by a versatile synthetic procedure. A comprehensive investigation reveals that the ChDT π‐core exhibits increasing structural stability in the bulk crystal phase, and that it is unaffected by a variation of the transfer integral, induced by the perpetual molecular motion of organic materials owing to the combination of its molecular shape and its particular HOMO configuration. Notably, ChDT derivatives exhibit excellent chemical and thermal stability, high charge‐carrier mobility under ambient conditions (μ ≤ 10 cm^2^ V^−1^ s^−1^), and a crystal phase that is highly stable, even at temperatures above 250 °C.

## 1 Introduction

Organic semiconductors are promising materials for the printed and flexible electronics such as organic field‐effect transistors (OFETs) due to several advantageous characteristics, which include solution processability, softness, and low weight.^1^ For the advancement of OFET‐based applications such as radio‐frequency identifier (RF‐ID) tags and sensors, the development of organic semiconductors with a charge‐carrier mobility (μ) of ≈10 cm^2^ V^−1^ s^−1^ is necessary. Unlike inorganic semiconductors, organic semiconductors generally engage in intermolecular interactions via noncovalent bonds based on van der Waals forces. To achieve high charge‐carrier mobility in organic semiconductors, the molecules should be densely packed in the solid state, which ensures effective overlap of the molecular orbitals between neighboring molecules. To date, promising semiconducting π‐electronic cores (π‐cores) have been reported, which include pentacene,^2^ [1]benzothieno[3,2‐*b*][1]benzothiophene (**BTBT**),^3^ dinaphtho[2,3‐*b*:2′,3′‐*f*]thieno[3,2‐*b*]thiophene (**DNTT**),^4^ dinaphtho[2,3‐*b*:2′,3′‐*d*]thiophene (**DNT–V**),^5^ and their derivatives. Their μ values exceed that of commonly used amorphous silicon (μ = 0.5–1.0 cm^2^ V^−1^ s^−1^). These molecules adopt a herringbone‐type packing structure, which is favorable for 2D charge‐carrier transport.^6^

The degree of molecular orbital overlap can be quantified theoretically as the transfer integral (*t*)^7^ and an effective molecular orbital overlap results in a large absolute value of *t*. Since aggregated structures of organic materials consist of weak intermolecular interactions, the molecules are perpetually in thermal motion, even in the solid state. However, thermal molecular motion might reduce the intermolecular orbital overlap and the thus decreased *t* values diminish μ. Therefore, the variation of the transfer integral (Δ*t*) due to molecular motion may be considered as a parameter that represents the deterioration of μ, which is of critical importance to thoroughly understand the underlying charge‐transporting properties toward the realization of increased μ values.^8^

**Figure 1:**
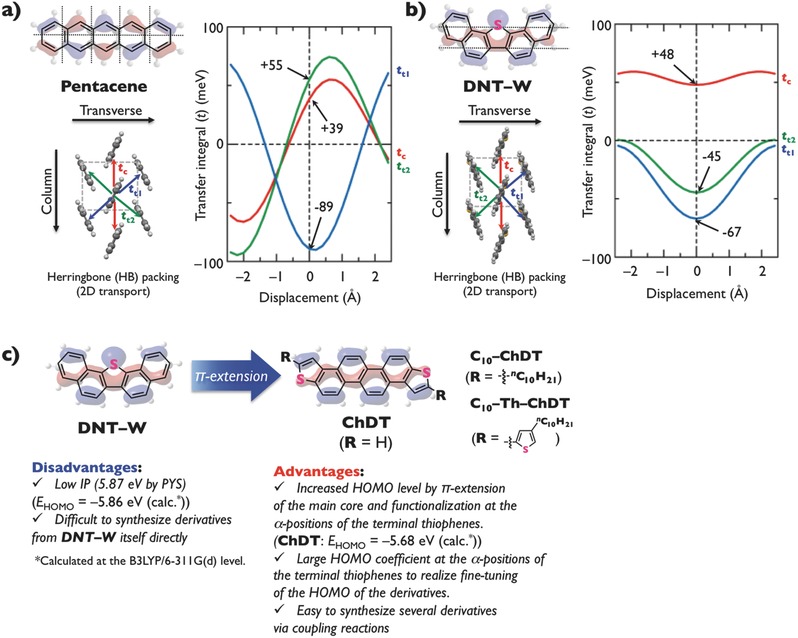
Chemical structures, HOMO configurations, and packing structures of a) pentacene and b) DNT–W with transfer integrals depending on the displacement from their original packing structures in the longitudinal molecular direction, calculated at the B3LYP/6‐31G(d) level of theory. c) Molecular design strategy for the zigzag‐shaped ChDT derivatives in this work.

For pentacene in a herringbone‐type packing structure,^9^ *t* values of +39, +55, and −89 meV have been calculated in the direction of the red, green, and blue arrows, respectively (**Figure** **1**a). The *t* values between two neighboring pentacene molecules can be estimated as a function of the displacement from the original position (displacement value = 0) along the longitudinal molecular axis. As the molecular motion tends to occur in the most prominent direction for compounds with herringbone‐type packing, we carried out a calculation along the molecular longitudinal axis.^10^ The *t* values of pentacene periodically change upon displacement in all directions, consistent with previously reported model calculations on the cofacial dimer of tetracene,^11^ which revealed alternating nodal changes of the pentacene highest occupied molecular orbital (HOMO) along the longitudinal and transversal molecular axes (Figure 1a). Other π‐cores such as **BTBT**, **DNTT**, and **DNT–V**, which also pack in a herringbone‐type packing structure, exhibit the same HOMO configuration (Figure S1, Supporting Information). Such a HOMO configuration may be disadvantageous for a versatile organic semiconducting π‐core for three main reasons: 1) only a precisely controlled displacement along both molecular axes in the packing structure may realize the effective orbital overlap necessary for large absolute *t* values; 2) the *t* values for these π‐cores periodically change upon displacement in all directions; and 3) the Δ*t* values are relatively large due to the fact that the perpetual molecular motion decreases μ.^12^

To circumvent these obstacles, we have focused on π‐cores with a specific HOMO configuration, in which the same phase extends along the longitudinal molecular axis. Recently, we have reported that W‐shaped dinaphtho[1,2‐*b*:2′,1′‐*d*]thiophene (**DNT–W**) π‐cores exhibit such a HOMO configuration (Figure 1b).^13^ Compared to pentacene, **DNT–W** exhibits larger absolute *t* values and much smaller Δ*t* values. Based on a theoretical comparison, molecules with such a HOMO configuration should exhibit high μ values due to the lower restrictions arising from displacement as well as molecular motion along the longitudinal molecular axis. Although previously reported **DNT–W** is promising in these respects, its π‐core exhibits several disadvantages, which require improvement: 1) the high ionization potential (*IP* = 5.87 eV), determined by photoelectron yield spectroscopy (PYS),^13^ causes the resulting transistor to operate at a large driving voltage due to the injection barrier from commonly used gold electrodes (4.9–5.0 eV); 2) the lack of synthetic methodologies to functionalize the terminal benzene rings of **DNT–W** with, e.g., alkyl substituents^14^ currently hampers the fine‐tuning of the HOMO level and the solution processability.

Herein, we report zigzag‐shaped chryseno[2,1‐*b*:8,7‐*b*′]dithiophene (**ChDT**) as a new semiconducting π‐core (Figure 1c). The molecular design strategy for **ChDT** is based on three main points: 1) the HOMO configuration of **ChDT**, which exhibits the same phase along the longitudinal molecular axis, is similar to that in **DNT–W**; 2) the calculated HOMO energy level of **ChDT** (*E*_HOMO_ = −5.68 eV) is significantly higher than that of **DNT–W** (*E*_HOMO_ = −5.86 eV),^15^ which should reduce the driving voltage in devices via its π‐conjugation extension. Furthermore, since the HOMO of **ChDT** possesses a large orbital coefficient at the α‐position of the terminal thiophene units, introducing an electron‐donating group should effectively elevate its energy level (Figure S2, Supporting Information); 3) the zigzag‐shaped **ChDT** skeleton may furthermore provide structural stability in the bulk crystal phase by suppressing molecular motion similar to V‐ and N‐shaped π‐cores previously reported by our group.^5^, ^16^ These structural features should also increase the thermal durability in field‐effect transistors (FETs). In this article, we discuss a versatile synthetic route to **ChDT** and its derivatives that contain either *n*‐decyl or *n*‐decylthienyl substituents at the α‐position of the terminal thiophene rings of **ChDT** (**C_10_–ChDT** and **C_10_–Th–ChDT**; Figure 1c). Their fundamental properties in solution and in thin films, their aggregation structures in single crystals, and their single‐crystal‐based transistors were investigated experimentally and theoretically. Notably, a solution‐crystallized FET based on **C_10_–Th–ChDT** exhibited a hole mobility of up to 10 cm^2^ V^−1^ s^−1^ under ambient conditions and a crystal phase that was stable at temperatures beyond 250 °C, prompting us to investigate applications based on printed and flexible organic electronics.

## 2 Results and Discussion

### 2.1 Synthesis and Fundamental Properties

**Figure 2:**
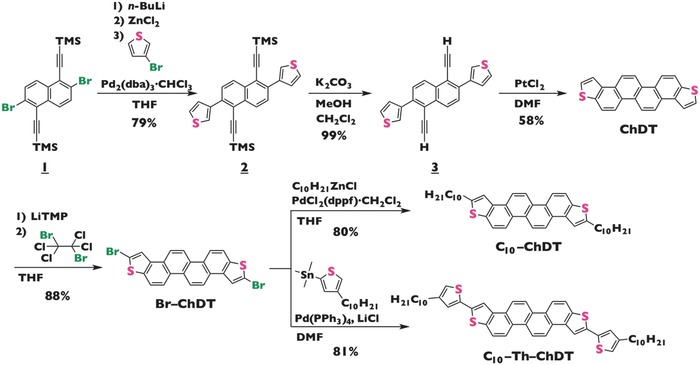
Synthetic route to the ChDT derivatives used in this study.

**Figure** **2** illustrates the synthetic route to **ChDT** and its derivatives. Compound **2** was obtained from consecutively subjecting 2,6‐dibromo‐1,5‐bis(trimethylsilylethynyl)naphthalene (**1**)^17^ to a halogen–metal exchange, a transmetallation, and a Negishi cross‐coupling reaction with 3‐bromothiophene. A subsequent deprotection of the trimethylsilyl groups, followed by a cyclization using a PtCl_2_ catalyst in toluene,^18^ furnished **ChDT** in low yield. To improve the yield, we screened other solvents, including polar solvents, such as *N*,*N*‐dimethylformamide and *N*‐methylpyrrolidone, which afforded **ChDT** in higher yield (≤58%). After a selective deprotonation of **ChDT** at the α‐position of the terminal thiophene units using lithium tetramethylpiperidide, a subsequent treatment with 1,1,2,2‐tetrachloro‐1,2‐dibromoethane^19^ furnished dibrominated **ChDT** (**Br–ChDT**) as a key precursor. Using either Negishi or Kosugi–Migita–Stille coupling reactions, **Br–ChDT** was treated in the presence of a palladium catalyst with *n*‐decyl zinc chloride or (4‐decylthiophen‐2‐yl)trimethylstannane, respectively, to afford the target compounds **C_10_–ChDT** (80%) and **C_10_–Th–ChDT** (81%) as white solids. All **ChDT** derivatives were purified by multiple recrystallization and gel permeation chromatography steps in order to obtain device‐grade samples.

Initially, we tested the solubility of **C_10_–ChDT** and **C_10_–Th–ChDT** in toluene at 60 °C. The results were compared to those of **C_10_–DNTT** 20, 21 and 3,11‐didecyldinaphtho[2,3‐*d*:2′,3′‐*d*′]benzo[1,2‐*b*:4,5‐*b*′]dithiophene (**C_10_–DNBDT–NW**)^16^ (Table S1, Supporting Information). The solubility of **C_10_–ChDT** (0.068 wt%) is approximately six‐ and twofold higher than that of **C_10_–DNTT** (0.010 wt%) and **C_10_–DNBDT–NW** (0.033 wt%), respectively. The introduction of thienyl groups resulted in a much higher solubility of **C_10_–Th–ChDT** (0.13 wt%) due to the degree of freedom between the thienyl groups and the **ChDT** π‐core. Next, the IP values were determined by PYS.^22^ The IP value of the parent **ChDT** (5.84 eV) is slightly smaller than that of **DNT–W** (5.87 eV). **C_10_–ChDT** and **C_10_–Th–ChDT** exhibit even smaller values of 5.56 eV and 5.53 eV, respectively, indicating that these **ChDT** derivatives should exhibit an improved injection of charge carriers from the commonly used gold electrodes (Figure S3, Supporting Information). Furthermore, the time‐dependent UV–vis absorption spectrum of π‐extended **C_10_–Th–ChDT** in solution under atmospheric conditions indicated high chemical stability, as the spectrum remained unchanged, even after two weeks (Figure S4, Supporting Information). These results indicate that all these **ChDT** derivatives are air‐stable organic semiconductors that can be handled without special care in solution or when using solution processes to fabricate devices.

The thermal properties of these **ChDT** derivatives were measured by means of thermogravimetric analysis (TGA) and differential scan calorimetry (DSC) (Figures S5 and S6, Supporting Information). Under a N_2_ atmosphere, the TGA revealed 5% weight‐loss temperatures (*T* ^95%^) of 379 and 428 °C for **C_10_–ChDT** and **C_10_–Th–ChDT**, respectively. The phase‐transition behavior and temperatures were determined by DSC. It is noteworthy that the obvious phase transitions from the initial crystal phases of **C_10_–ChDT** and **C_10_–Th–ChDT** occur above 280 and 254 °C, respectively; these values are extremely high compared to other linear‐shaped π‐cores such as decyl‐substituted pentacene, **BTBT**, and **DNTT** (cf. C_10_–pentacene: 89 °C; **C_10_–BTBT**: 110 °C; **C_10_–DNTT**: 108 °C).^23^, ^24^, ^25^ Interestingly, **ChDT** derivatives exhibit more promising thermally stabilized crystal phases than the previously reported V‐shaped **C_10_–DNT–VW** (150 °C) and the N‐shaped **C_10_–DNBDT–NW** (217 °C).^5^, ^16^ Thus, replacing and extending the bent π‐cores of V‐ and N‐shaped derivatives with a zigzag‐type **ChDT** core affords a thermally stabilized crystal phase. The thermal stress test of a device including a **ChDT**‐based transistor will be reported elsewhere.

### 2.2 Crystal Structures

**Figure 3:**
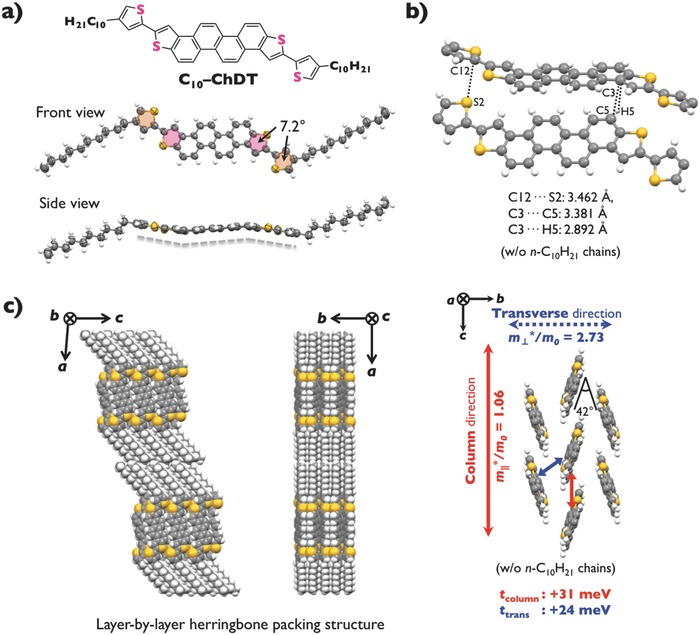
a) Chemical and molecular structure of **C_10_–Th–ChDT** in the single crystal. b) Intermolecular interaction and short contacts of **C_10_–Th–ChDT**. c) Packing structure of **C_10_–Th–ChDT** in the single crystal, together with theoretical calculations regarding the transfer integrals and effective mass values based on the coordinates of **C_10_–Th–ChDT**.

To unambiguously determine the molecular and packing structures of the **ChDT** derivatives, as well as to calculate their charge‐transporting properties using their coordinates, single‐crystal X‐ray diffraction (XRD) analyses were carried out. Single crystals of the parent **ChDT** were grown by the physical vapor transport (PVT) technique.^26^, ^27^ Single crystals of soluble **C_10_–ChDT** and **C_10_–Th–ChDT** were grown by gradual diffusion of isopropanol into toluene solutions of the target compounds. All derivatives yielded sheet‐like single crystals, which were used in the X‐ray diffraction measurements. The molecular and packing structures of **ChDT**, **C_10_–ChDT**, and **C_10_–Th–ChDT** are summarized in Figures S7–S12 (Supporting Information), **Figure** **3**, and Table S2 (Supporting Information). As shown in Figure 3, the **ChDT** unit in the **C_10_–Th–ChDT** molecule exhibits a bent conformation (Figure 3a, side view), which is similar to those of decyl‐substituted V‐ (**C_10_–DNT–VW**) and N‐shaped molecules (**C_10_–DNBDT–NW**).^5^, ^16^ A dihedral angle of 7.2° was observed between the decylthienyl and the terminal thiophene moieties of the **ChDT** π‐core of **C_10_–Th–ChDT**. Even though all **ChDT** derivatives form 2D ordered herringbone structures, tilt angles of 60°, 89°, and 42° were observed for **ChDT**, **C_10_–ChDT**, and **C_10_–Th–ChDT**, respectively (Figures S9 and S12 in the Supporting Information and Figure 3c). These herringbone packing structures exhibit two predominant types of short C–H⋯π and S⋯π interactions (Figures S8 and S11 in the Supporting Information and Figure 3b), which are shorter than the sum of van der Waals radii for hydrogen (1.20 Å), carbon (1.70 Å), and sulfur (1.85 Å).^28^

### 2.3 Theoretical Calculations

Based on the packing structures of the **ChDT** derivatives, we calculated their *t* values to investigate their potential as semiconducting materials. The *t* values of the HOMOs between neighboring molecules were estimated by the dimer method based on density functional theory calculations at the PBEPBE/6‐31G(d) level of theory.^7^ The parent **ChDT** exhibits *t* values of +73 meV and −63 meV in the column and the transverse directions, respectively (Figure S13, Supporting Information). In the case of **C_10_–ChDT** with a lying herringbone packing structure (tilt angle: 89°; Figure S12, Supporting Information), the estimated *t* values are −66 meV (column) and +22 meV (transverse). On the other hand, the packing structure of **C_10_–Th–ChDT** (tilt angle: 42°; Figure 3c) exhibits smaller absolute and positive *t* values of +31 meV (column) and +24 meV (transverse), respectively (Figure 3c). Although **C_10_–Th–ChDT** packs with a non‐negligible displacement of 1.51 Å in the longitudinal molecular axis (Figure S14, Supporting Information), the different trends regarding the *t* values between these molecules should be attributed mainly to the intermolecular distances and the tilt angles rather than to the molecular displacement in the herringbone packing structure, because the transfer integral, as illustrated in the **ChDT** cases, is not very susceptible to the displacement parameter. Thus, the main reason for the smaller absolute *t* values of **C_10_–Th–ChDT** is that the π–π stacking distance of **C_10_–Th–ChDT** (6.91 Å) is much longer than that of **ChDT** (5.97 Å), due to the steric hindrance of the decylthienyl groups in **C_10_–Th–ChDT**.

To better understand the carrier‐transporting properties in the bulk, we calculated the electronic band structure at the same level as the intermolecular electronic coupling, using the periodic boundary condition at the PBEPBE/6‐31G(d) level of theory (Figures S15–17, Supporting Information). Generally, μ is inversely proportional to the effective mass (m*) according to

(1) $$\mu =q\tau /m^{*}$$

wherein μ refers to the mobility, *q* to the elementary charge, τ to the relaxation time, and m* to the effective mass.^29^

When the tight‐binding approximation is applied to a 2D ordered herringbone packing structure, m* is inversely proportional to the square of the distance between the intermolecular centroids. **Table** **1** summarizes the calculated m* values for the **ChDT** derivatives together with those of other reported semiconductor, **C_10_–DNBDT–NW**,^16^ using their single‐crystal X‐ray diffraction data. All semiconductors exhibit anisotropic values, i.e., the effective mass in the column direction (*m*_||_*) is smaller than that in the transverse direction (*m*_⊥_*) (Figure 3c and Table 1), indicating a high carrier mobility in the column direction. Notably, the *m*_||_* of **C_10_–Th–ChDT** (*m*_||_* = 1.06 *m*_0_) is much smaller than that of **ChDT** (1.19 *m*_0_), while the absolute *t* values of **C_10_–Th–ChDT** are smaller than those of **ChDT**. Furthermore, the *m*_||_* value of **C_10_–Th–ChDT** (1.06 *m*_0_) is comparable to that of **C_10_–DNBDT–NW** (*m*_||_* = 1.05 *m*_0_), which exhibits a high carrier mobility (μ ≤ 16 cm^2^ V^−1^ s^−1^).^16^ Accordingly, the results of the band calculations indicate that the increase of the distance between the intermolecular centroids of **C_10_–Th–ChDT** compensates for the reduction of the transfer integral, resulting in a small effective mass.

**Table 1:** Effective mass and mobility values for the ChDT derivatives together with C_10_–DNBDT–NW (L: lamination method; EC: edge‐casting method)

	Effective mass		
Material	m*_||_/*m*_0_	m*_⊥_/*m*_0_	Mobility [cm^2^ V^−1^ s^−1^]
**ChDT**	1.19	1.80	3.1(L)
**C_10_–ChDT**	2.02	9.00	2.6 (EC)
**C_10_–Th–ChDT**	1.06	2.73	10 (EC)
**C_10_–DNBDT–NW**	1.05	2.11	16 (EC)

**Figure 4:**
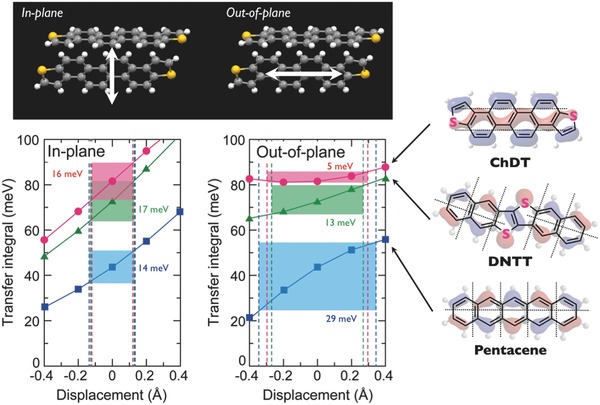
Transfer integral values (*t*) and their variations (Δ*t*) for **ChDT** in the in‐plane and out‐of‐plane directions, as well as the corresponding values for pentacene and DNTT for comparison.

Furthermore, to clarify and demonstrate the specific HOMO configuration of the **ChDT** π‐core, the relationship between the displacement and the transfer integrals, based on the packing structure of **ChDT** as well as those of pentacene and **DNTT** for comparison, was theoretically calculated in the in‐plane and out‐of‐plane directions (**Figure** **4**). In both directions, the transfer integrals of **ChDT** are larger than those of pentacene and **DNTT**. Since organic molecules are perpetually in thermal motion due to their weak intermolecular interaction, even in single crystals, Δ*t* is a critically important parameter toward the realization of longer relaxation times (τ) (cf. Equation (1)). It should be noted that in terms of the out‐of‐plane motion, which is the most prominent direction of molecular vibrations in the herringbone packing,^10^ the Δ*t* values of **ChDT** (5 meV) are apparently smaller than those of pentacene (29 meV) and **DNTT** (13 meV) in the range of 0.13–0.18 Å (Figure S18 and Table S3, Supporting Information), while the Δ*t* values of all compounds are comparable in the in‐plane motion. Our theoretical calculations thus indicate that molecules with the same phase along the longitudinal molecular axis should be promising prospectives for next‐generation organic semiconductors.

### 2.4 Device Evaluations

**Figure 5:**
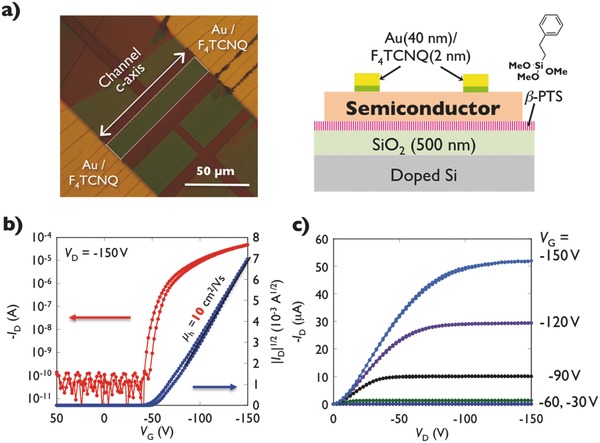
a) Device structure and microscopic images. b) Transfer characteristics of solution‐crystallized thin films of **C_10_–Th–ChDT**. c) Output characteristics for devices based on solution‐crystallized thin films of **C_10_–Th–ChDT**.

Finally, the μ values of the **ChDT** derivatives were evaluated in single‐crystal‐based FETs, which reveals the intrinsic carrier transport properties because the parasitic effect and extrinsic factors such as grain boundaries can be neglected.^30^, ^31^, ^32^ Typical transistor performances are shown in Figure S20 (Supporting Information) (**ChDT**), Figure S21 (Supporting Information) (**C_10_–ChDT**), and **Figure** **5**b,c (**C_10_–Th–ChDT**). All derivatives show p‐type behavior with a negligible hysteresis against the *V*_G_ sweep. Single crystals of **ChDT** show a high carrier mobility (μ = 3.1 cm^2^ V^−1^ s^−1^), whereby the threshold voltage (−25 V) improves compared to that of **DNT–W** (–50 V) in the same fluorinated decylsilyl self‐assembled monolayer (FDTS‐SAM)‐treated SiO_2_/Si substrates and same device configuration. The FDTS‐SAM can act as avoiding contamination adhesion onto the surface and positively shifting the threshold voltage (*V*_th_) by its dipole moment.^33^ In the case of single‐crystalline thin films of **C_10_–Th–ChDT** and **C_10_–ChDT**, the former exhibits a higher mobility (μ = 10 cm^2^ V^−1^ s^−1^) in the saturation regime than the latter (μ = 2.6 cm^2^ V^−1^ s^−1^). These carrier mobility results are consistent with the trends observed in the theoretical calculations (Table 1). In terms of threshold voltages of **C_10_–Th—ChDT‐** and **C_10_–ChDT‐** based transistor, much smaller values of –50 V and –100 V were observed, because phenyl‐terminated SAM SiO_2_/Si substrate was applied to increase the wettability of commonly used aromatic solvents in various solution processes. To clarify the crystalline film morphology and channel directions for the best semiconductors of **C_10_–Th–ChDT**, from the results of atomic force microscopy (AFM) images in Figure S23 (Supporting Information), the steps correspond to two‐molecular step‐height of **C_10_–Th–ChDT** and smooth crystal surface were observed, indicating that well‐oriented crystalline films are grown. Furthermore, transmission XRD measurements in both out‐of‐plane and in‐plane directions were performed. The out‐of‐plane XRD data revealed that the *a*‐axis, which corresponds to the longitudinal molecular axis, is oriented perpendicular relative to the substrate, while the *bc* conduction plane is arranged in parallel relative to the substrate. The results of the in‐plane XRD measurements suggested that the column direction of the *c*‐axis, which exhibits the smaller effective mass, is arranged almost in parallel with respect to the crystal growth direction (Figure S24) (Supporting Information).

## 3 Conclusion

Using a versatile synthetic procedure, we synthesized several zigzag‐shaped **ChDT** derivatives as new semiconducting π‐cores. The derivatives exhibit a peculiar HOMO configuration that differs from those of conventional π‐cores of fused acenes and heteroacenes with high carrier mobilities. In the solid state, all these **ChDT** derivatives adopt herringbone‐type packing structures with different tilt angles. Band calculations based on these packing structures indicated that **C_10_–Th–ChDT** exhibits the smallest effective mass among these derivatives. Theoretical investigations revealed that such a HOMO configuration is advantageous, as the structure is not susceptible to variations of the transfer integral, which inhibits the molecular displacement in aggregates, induced by the perpetual molecular motion of organic materials. Furthermore, the zigzag‐shaped feature effectively stabilizes the crystal phase, even at temperatures beyond 250 °C, which should allow a relatively easy production of **ChDT**‐based devices with high durability toward thermal stress. Notably, **ChDT** derivatives exhibit excellent chemical and thermal stability, as well as a high carrier mobility (μ ≤ 10 cm^2^ V^−1^ s^−1^) in single‐crystalline thin films under ambient conditions. A further examination of the optimization of the solubility and the crystallinity may lead to further improved carrier‐transport abilities. An extensive investigation into **ChDT** derivatives and their congeners is currently underway in our laboratory.

## 4 Experimental Section

*General for Characterization*: All NMR spectra were recorded on an ECS400 spectrometer. Mass spectra were measured on a BRUKER compact‐TKP2 mass spectrometer. Melting points and elemental analysis were collected on a Mettler Toledo MP70 Melting Point System and J‐Science Lab JM10 MICRO CORDER, respectively. Photoelectron yield spectroscopy was performed on a Sumitomo Heavy Industries Advanced Machinery PYS‐202. UV–vis absorption was measured with a JASCO V‐570 spectrometer. TGA and DSC measurements were carried out with a Rigaku Thermo Plus EVO II TG 8121 and a Rigaku Thermo Plus EVO IIDSC 8231, respectively. Single‐crystal diffraction data were collected on a Rigaku R‐AXIS RAPID II imaging plate diffractometer with CuKα radiation. AFM was carried out with a Shimadzu SPM‐9700HT. Transistor characterizations were carried out using a Keithley 4200 semiconductor parameter analyzer.

*Compound Data: **ChDT***: ^1^H NMR (400 MHz, TCE‐*d*_2_): δ 7.67(d, 2H, *J* = 5.6 Hz, Ar*H*), 8.11 (d, 2H, *J* = 5.6 Hz, Ar*H*), 8.14 (d, 2H, *J* = 9.2 Hz, Ar*H*), 8.56 (d, 2H, *J* = 9.2 Hz, Ar*H*), 8.75 (d, 2H, *J* = 9.2 Hz, Ar*H*), and 8.91 (d, 2H, *J* = 9.2 Hz, Ar*H*). ^13^C‐NMR could not be recorded due to the poor solubility. Anal. Calcd for C_22_H_12_S_2_: C 77.61; H 3.55. Found: C 77.25; H 3.55. TOF HRMS (APCI+): Calcd for C_22_H_13_S_2_ [M+H] 341.0459. Found: 341.0443.

***C_10_–ChDT***: m.p.: 315−317 °C. ^1^H NMR (400 MHz, TCE‐*d*_2_): δ 0.90 (t, 6H, *J* = 6.0 Hz, C*H_3_*), 1.20−1.60 (m, 28H, C*H_2_*), 1.79−1.92 (m, 4H, Ar‐CH_2_‐C*H_2_*), 3.06 (t, 4H, *J* = 7.6 Hz, Ar‐C*H_2_*), 7.76 (s, 2H, β position of the thiophene ring), 8.02 (d, 2H, *J* = 9.2 Hz, Ar*H*), 8.47 (d, 2H, *J* = 9.2 Hz, Ar*H*), 8.64 (d, 2H, *J* = 9.2 Hz, Ar*H*), and 8.84 (d, 2H, *J* = 8.8 Hz, Ar*H*). ^13^C NMR (TCE‐*d*_2_): δ 14.0, 22.6, 29.2, 29.2, 29.4, 29.5, 29.6, 31.1, 31.4, 31.9, 118.9, 119.0, 121.1, 121.9, 123.3, 127.2, 127.8, 128.3, 137.2, 137.3, and 147.7. TOF HRMS (APCI+): Calcd for C_42_H_53_S_2_ [M+H] 621.3589, found, 621.3598. Anal. Calcd for C_42_H_52_S_2_: C 80.23; H 8.44. Found: C 80.12; H 8.27.

***C_10_–Th–ChDT***: m.p.: 307−309 °C. ^1^H NMR (400 MHz, TCE‐*d*_2_): δ 0.88 (t, 6H, *J* = 6.6 Hz, C*H_3_*), 1.20−1.50 (m, 28H, C*H_2_*), 1.67 (quin, 4H, *J* = 7.4 Hz, Ar‐CH_2_‐C*H_2_*), 2.64 (t, 4H, *J* = 7.4 Hz, Ar‐C*H_2_*), 6.94 (s, 2H, Ar*H* of thienyl group), 7.23 (s, 2H, Ar*H* of thienyl group), 8.04 (d, 2H, *J* = 9.2 Hz, Ar*H*), 8.10 (s, 2H, Ar*H*), 8.52 (d, 2H, *J* = 9.2 Hz, Ar*H*), 8.70 (d, 2H, *J* = 9.2 Hz, Ar*H*), and 8.86 (d, 2H, *J* = 9.2 Hz, Ar*H*). ^13^C NMR (TCE‐*d*_2_): δ 14.0, 22.7, 29.2, 29.3, 29.4, 29.5, 29.6, 30.3, 31.5, 31.9, 117.5, 120.0, 121.0, 122.1, 123.4, 125.1, 125.2, 127.3, 128.0, 128.5, 137.8, 137.1, 137.6, 138.6, and 147.2. TOF HRMS (APCI+): Calcd for C_50_H_57_S_4_ [M+H] 785.3343. Found: 785.3366. Anal. Calcd for C_50_H_56_S_4_: C 76.48; H 7.19. Found: C 76.55; H 7.11.

*Device Fabrication*: The FET fabrication and measurements were carried out under ambient conditions. For insoluble **ChDT**, a platelet single crystal grown by the PVT technique^26^, ^27^ was manually laminated on fluorinated decylsilyl self‐assembled monolayer‐treated SiO_2_/Si substrates.^33^ The thus obtained results can hence be directly compared to previously reported **DNT−W** data.^13^ On the other hand, for **C_10_−ChDT** and **C_10_−Th−ChDT**, which are soluble in organic solvents, single‐crystalline thin films were grown on phenylethylsilyl self‐assembled monolayer (β‐PTS‐SAM)‐treated SiO_2_/Si substrates using the edge‐casting method (Figure S19, Supporting Information).^34^ The β‐PTS‐SAM treatment increases the wettability for commonly used aromatic organic solvents to reproducibly obtain single‐crystalline films.^35^ On top of the single crystals, an F_4_–TCNQ hole injection layer, as well as gold source and drain electrodes were successively deposited through a shadow mask to construct top‐contact‐bottom‐gate configurations (Figure 5a).^36^

[CCDC 1544287–1544289 contains the supplementary crystallographic data for this paper. These data can be obtained free of charge from The Cambridge Crystallographic Data Centre via www.ccdc.cam.ac.uk/data_request/cif.]

## Conflict of Interest

The authors declare no conflict of interest.

## Supporting information

Supplementary
