# Supplementary material for: Zigzag‐Elongated Fused π‐Electronic Core: A Molecular Design Strategy to Maximize Charge‐Carrier Mobility
Source: Adv Sci (Weinh). 2017 Nov 15;5(1):1700317. doi: 10.1002/advs.201700317 (PMC5770660; doi:10.1002/advs.201700317)
Supplement: Supplementary file 1 — Supplementary [file ADVS-5-na-s001.pdf]

Copyright WILEY-VCH Verlag GmbH & Co. KGaA, 69469 Weinheim, Germany, 2017.

# Supporting Information

for *Adv. Sci.,* DOI: 10.1002/advs.201700317

## Zigzag-Elongated Fused #-Electronic Core: A Molecular Design Strategy to Maximize Charge-Carrier Mobility

*Akito Yamamoto, Yoshinori Murata, Chikahiko Mitsui, Hiroyuki Ishii,* Masakazu Yamagishi, Masafumi Yano, Hiroyasu Sato, Akihito Yamano, Jun Takeya, and Toshihiro Okamoto**

WILEY-VCH

Copyright WILEY-VCH Verlag GmbH & Co. KGaA, 69469 Weinheim, Germany, 2016.

# Supplementary Information

## Zigzag-Elongated Fused π-Electronic Core: A Molecular Design Strategy To Maximize Charge Carrier Mobility

*Akito Yamamoto,[1] Yoshinori Murata,[2] Chikahiko Mitsui,[1] Hiroyuki Ishii,[3,*] Masakazu Yamagishi,[4] Masafumi Yano,[2] Hiroyasu Sato,[5] Akihito Yamano,[5] Jun Takeya,[1] Toshihiro Okamoto[1,6,*]*

*[1] Department of Advanced Materials Science, Graduate School of Frontier Sciences, The University of Tokyo, 5-1-5 Kashiwanoha, Kashiwa, Chiba 277-8561, Japan*

*[2] Chemistry, Materials and Bioengineering Major, Graduate School of Science and Engineering, Kansai University, 3-3-35 Yamate-cho, Suita, Osaka 564-8680, Japan*

*[3] Division of Applied Physics, Faculty of Pure and Applied Sciences, University of Tsukuba, 1-1-1 Tennodai, Tsukuba, Ibaraki 305-8573, Japan*

*[4] National Institute of Technology, Toyama College, 13 Hongo-machi, Toyama, Toyama 939-8630, Japan*

*[5] Rigaku Corp., 3-9-12 Matsubara-cho, Akishima, Tokyo 196-8666, Japan*

*[6] PRESTO, JST, 4-1-8 Honcho, Kawaguchi, Saitama 332-0012, Japan*

*Correspondence and requests for materials should be addressed to tokamoto@edu.k.u-tokyo.ac.jp or ishii@bk.tsukuba.ac.jp

# Contents


1. **Synthesis**
2. **Theoretical Predictions**
3. **Solubility Test**
4. **Ionization Potentials**
5. **Chemical Stability Test**
6. **Thermal Analyses**
7. **Single-Crystal Analyses**
8. **Transfer Integral and Band Calculations**
9. **Calculations for Amplitude of Translational Motions**
10. **OFET Device Fabrication and Evaluation Procedure**
11. **Atomic Force Microscopy**
12. **X-ray-diffraction Measurements for Solution-crystallized Thin Film**
13. **References**

# 1. Synthesis

## 1.1 Materials

Reagents and Starting Materials

2,6-Dibromo-1,5-dihydroxynaphthalne, trifluoromethanesulfonic anhydride, trimethylsilylacetylene, diisopropylamine, 3-bromothiophene, 1,2-dibromo-1,1,2,2-tetrachloroethane and 2,2,6,6-tetramethylpiperidine were purchased from Tokyo Chemical Industry Co., Ltd. Pyridine, $PdCl_2$(dppf)· $CH_2Cl_2$ and potassium carbonate were purchased from Wako Pure Chemical Industries, Ltd. Zinc chloride, $Pd_2(dba)_3$· $CHCl_3$, Sphos, $PtCl_2$, all Grinard reagents and LiCl in THF solution were purchased from Sigma-Aldrich Inc. *n*-BuLi and all anhydrous solvents were purchased from KANTO chemical Co., Ltd.

Preparation of zinc chloride solution

An oven-dried Schlenk tube was charged with zinc chloride (479.5 mg, 3.50 mmol), and the tube was heated under evacuation until zinc chloride completely melted. After cooled to room temperature, THF (3.5 mL) was added to give 1.0 M zinc chloride solution in THF.

Preparation of LiTMP

To a solution of 2,2,6,6-tetramethylpiperidine (TMP, 1.54 mL, 9.0 mmol) in THF (11.0 mL) was added *n*-BuLi (1.60 M hexane solution, 5.60 mL, 9.0 mmol) at –78 °C. After stirred at 0 °C for 30 min, 0.50 M LiTMP solution was obtained.

## 1.2 Methods

General for Synthesis and Characterization

All the reactions were carried out under an atmosphere of argon. Air- or moisture-sensitive liquids and solutions were transferred via a syringe or a Teflon cannula. Analytical thin-layer chromatography (TLC) was performed on glass plates with 0.25 mm 230–400 mesh silica gel containing a fluorescent indicator (Merck Silica gel 60 F254). TLC plates were visualized by exposure to ultraviolet lamp (254 nm and 365 nm) and by dipping with 10% phosphomolybdic acid in ethanol and heating on a hot plate. Flash column chromatography was performed on Kanto silica gel 60. Open column chromatography was performed on Wakogel C-200 (75–150 μm). All NMR spectra were recorded on a ECS400 spectrometer. Chemical shifts are reported in parts per million (ppm, $\delta$ scale) from residual protons in the deuterated solvent for $^1$H NMR ($\delta$ 7.26 ppm for chloroform, 5.93 ppm for 1,1,2,2-tetrachloroehtane (TCE)) and from the solvent carbon for $^{13}$C NMR ($\delta$ 77.16 ppm for

chloroform, 74.00 ppm for 1,1,2,2-tetrachloroehtane). The data were presented in the following format: chemical shift, multiplicity (s = singlet, d = doublet, t = triplet, m = multiplet), coupling constant in Hertz (Hz), signal area integration in natural numbers, assignment (italic). Mass spectra were measured on a BRUKER compact-TKP2 mass spectrometer. Melting points and elemental analysis were collected on a Mettler Toledo MP70 Melting Point System and J-Science Lab JM10 MICRO CORDER, respectively.

## 1.3 Experimental Procedure

### 2,6-Dibromo-1,5-bis(trifluoromethansulfonyl)naphthalene

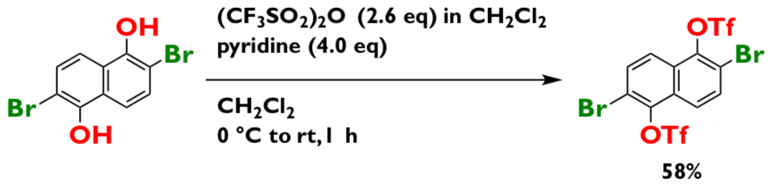


To a brown suspension of 2,6-dibromo-1,5-hydroxynaphthalene (48.9 g, 154 mmol, 1.00 mol amt.) in dichloromethane (310 mL) and pyridine (50.0 mL, 49.1 g, 621 mmol, 4.0 mol amt.) was added triflic anhydride (65.0 mL, 112 g, 396 mmol, 2.6 mol amt.) in dichloromethane (135 mL) dropwise at 0 °C. After the resulting black suspension was kept stirring at room temperature for an hour, the reaction was quenched by water. The organic layer was separated, and the aqueous layer was extracted with chloroform. The organic layer was washed with water three times, dried over $MgSO_4$. The resulting mixture was passed through a short pad of silica gel, and the solvent was evaporated *in vacuo*. The obtained black solid was purified by flush silica gel column chromatography (hexane: $CH_2Cl_2$ = 90:10 to 70:30) followed by recrystallization with hexane and chloroform (ca. 10:1) to afford the titled compound as a white solid (51.7 g, 58% yield). $^1$H NMR (400 MHz, CDCl3): $\delta$ 7.88 (d, 2H, $J$ = 8.8 Hz, Ar$H$), 8.02 (d, 2H, $J$ = 8.8 Hz, Ar$H$). This spectral data was in good agreement with the reported one.[S1]

### ((2,6-Dibromonaphthalene-1,5-diyl)bis(ethyne-2,1-diyl))bis(trimethylsilane) (1)

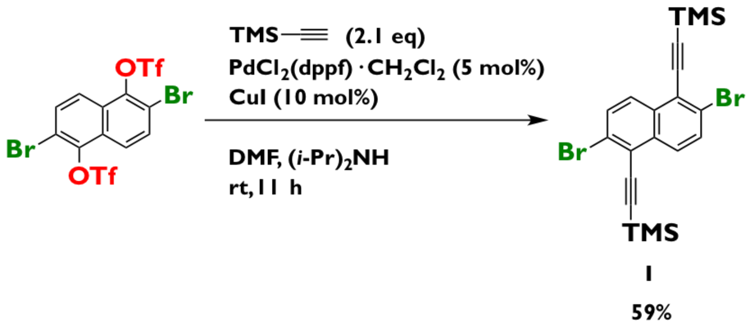

A colorless solution of 2,6-dibromo-1,5-bis(trifluoromethansulfonyl)naphthalene (46.72 g, 80.3 mmol, 1.00 mol amt.) in DMF (320 mL), diisopropylamine (320 mL) was bubbled by argon gas for 15 min to remove dissolved oxygen. To the solution was successively added CuI (1.52 g, 7.99 mmol, 10 mol%), $PdCl_2$(dppf)·$CH_2Cl_2$ (3.27 g, 4.00 mmol, 5.0 mol%), and trimethylsilylacetylene (23.5 mL, 16.7 g, 170 mmol, 2.1 mol amt.) at room temperature and the resulting black suspension was kept stirring at room temperature for 11 h. The resulting black suspension was dissolved in chloroform and passed through a short pad of silica gel to remove the inorganic salts. The crude material was purified by flush silica gel chromatography (using hexane as an eluent) to afford the titled compound as a white solid (22.46 g, 59% yield). $^1$H NMR (400 MHz, $CDCl_3$): $\delta$ 0.35(s, 18H, Si(C$H_3$)$_3$), 7.71 (d, 2H, $J$ = 8.8 Hz, Ar$H$), 8.14 (d, 2H, $J$ = 8.8 Hz, Ar$H$). This spectral data was in good agreement with the reported one.[S1]

**((2,6-Di(thiophen-3-yl)naphthalene-1,5-diyl)bis(ethyne-2,1-diyl))bis(trimethylsilane) (2)**

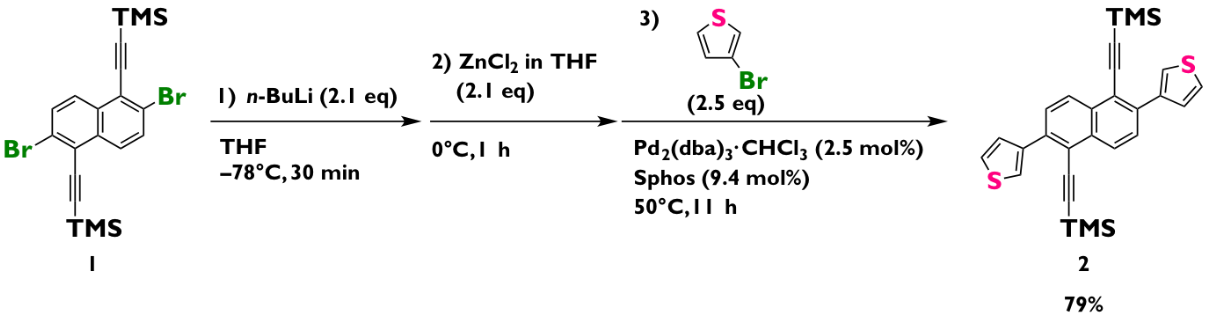


To a white suspension of **1** (574 mg, 1.20 mmol, 1.00 mol amt.) in THF (4.8 mL) was added $n$-BuLi in $n$-hexane (1.55 M, 1.60 mL, 2.48 mmol, 2.1 mol amt.) dropwise at –78°C and the resulting yellow suspension was kept stirring for 30 min at that temperature. To the yellow suspension was added $ZnCl_2$ in THF (1.0 M, 2.50 mL, 2.50 mmol, 2.1 mol amt.) at –78 °C. After stirring at 0 °C for 1 h, to the resulting pale yellow solution was added 3-bromothiophene (0.28 mL, 482 mg, 2.95 mmol, 2.5 mol amt.), $Pd_2(dba)_3$·$CHCl_3$ (30.5 mg, 0.029 mmol, 2.5 mol%), and SPhos (46.3 mg, 0.113 mmol, 9.4 mol%) at 0 °C. The resulting brown suspension was kept stirring at 50 °C for 11 h. After cooling to room temperature, suspension was passed through a short pad of silica gel, and evaporated *in vacuo*. The crude material was purified by flush silica gel chromatography (hexane: $CH_2Cl_2$ = 100:0 to 90:10) to yield the titled compound as a white solid (461 mg, 79%). m.p.: 191–194 °C. $^1$H NMR (400

MHz, $CDCl_3$): $\delta$ 0.29 (s, 18H, Si(C*H*$_3$)$_3$), 7.38–7.41 (m, 2H, Ar*H* of thienyl group), 7.62–7.65 (m, 2H, Ar*H* of thienyl group), 7.71 (d, 2H, *J* = 8.4 Hz, Ar*H* of naphthalene), 7.83–7.85 (m, 2H, Ar*H* of thienyl group), 8.45 (d, 2H, *J* = 8.4 Hz, Ar*H* of naphthalene). $R_f$ = 0.48 (eluent: hexane: $CH_2Cl_2$ = 80:20). $^{13}$C NMR ($CDCl_3$): $\delta$ –0.01, 102.7, 104.6, 117.8, 124.7, 124.8, 127.8, 128.4, 129.1, 132.9, 137.5, 140.9. TOF HRMS (APCI+): Calcd for $C_{28}H_{29}S_2Si_2$ [M+H] 485.1249. Found, 485.1270. Anal. Calcd for $C_{28}H_{28}S_2Si_2$: C 69.37; H 5.82. Found: C 69.28; H 5.87.

**3,3'-(1,5-Diethynylnaphthalene-2,6-diyl)dithiophene (3)**

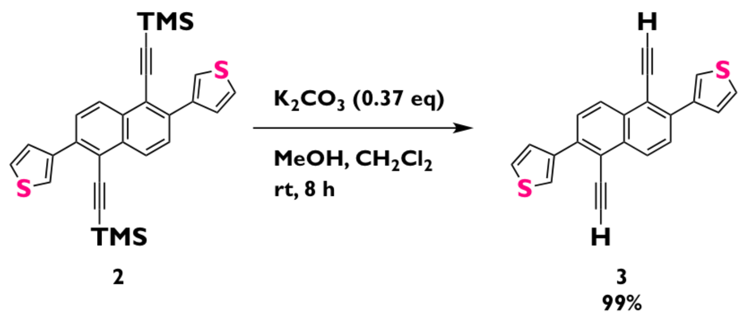


To a colorless solution of **2a** (206.0 mg, 0.425 mmol, 1.00 mol amt.) in MeOH (1.0 mL) and $CH_2Cl_2$ (3.0 mL) was added potassium carbonate (22.0 mg, 0.159 mmol, 0.37 mol amt.) at room temperature. After the resulting white suspension was kept stirring at that temperature for 8 h, the reaction mixture was quenched with water and extracted with chloroform. The organic layer was washed by water three times and volatiles were evaporated *in vacuo* to afford the titled compound as a white solid (143.2 mg, 99% yield). m.p.: 193–195 °C. $^1$H NMR (400 MHz, $CDCl_3$): $\delta$ 3.63 (s, 2H, *H* of terminal alkyne), 7.41–7.44 (m, 2H, Ar*H* of thienyl group), 7.60–7.62 (m, 2H, Ar*H* of thienyl group), 7.71 (d, 2H, *J* = 8.4 Hz, Ar*H* of naphthalene), 7.80–7.82 (m, 2H, Ar*H* of thienyl group), 8.52 (d, 2H, *J* = 8.4 Hz, Ar*H* of naphthalene). $^{13}$C NMR ($CDCl_3$): $\delta$ 81.2, 86.8, 117.0, 124.9, 125.1, 127.9, 128.6, 129.0, 133.2, 138.1, 140.8. TOF HRMS (APCI+): Calcd for $C_{22}H_{13}S_2$ [M+H] 341.0459. Found, 341.0473. Anal. Calcd for $C_{22}H_{12}S_2$: C 77.61; H 3.55. Found: C 77.54; H 3.64.

**Chryseno[2,1-*b*:8,7-*b*']dithiophene (ChDT)**

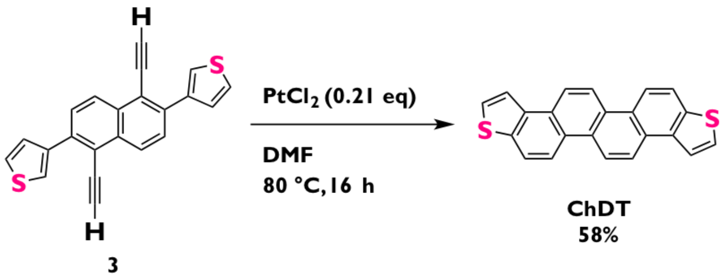


To a colorless solution of **3** (1.03 g, 3.02 mmol, 1.00 mol amt.) in DMF (30 mL) was added $PtCl_2$ (166 mg, 0.624 mmol, 0.21 mol amt.) at room temperature, and the resulting brown suspension was kept stirring at 80 °C for 16 h. After the reaction mixture was cooled to room temperature, the brown solid was filtered. The solid was dissolved with *o*DCB (ca. 1.5 L, 120 °C) and the resulting solution was passed through a short pad of silica gel. The resulting solution was condensed *in vacuo* and the solid was recrystallized from the solution to afford the titled compound as a yellow solid (593 mg, 58% yield). $^1$H NMR (400 MHz, TCE-$d_2$): $\delta$ 7.67 (d, 2H, $J$ = 5.6 Hz, Ar*H*), 8.11 (d, 2H, $J$ = 5.6 Hz, Ar*H*), 8.14 (d, 2H, $J$ = 9.2 Hz, Ar*H*), 8.56 (d, 2H, $J$ = 9.2 Hz, Ar*H*), 8.75 (d, 2H, $J$ = 9.2 Hz, Ar*H*), 8.91 (d, 2H, $J$ = 9.2 Hz, Ar*H*). $^{13}$C NMR could not be recorded due to the poor solubility. TOF HRMS (APCI+): Calcd for $C_{22}H_{13}S_2$ [M+H] 341.0459. Found, 341.0443. Anal. Calcd for $C_{22}H_{12}S_2$: C 77.61; H 3.55. Found: C 77.25; H 3.55.

**2,9-Dibromochryseno[2,1-*b*:8,7-*b*']dithiophene (Br–ChDT)**

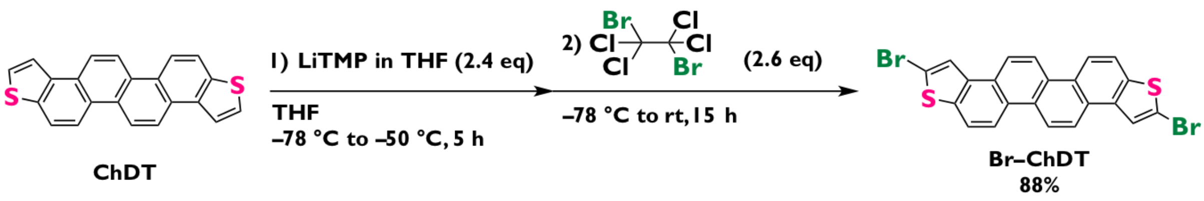


To a brown suspension of **4a** (2.370 g, 6.96 mmol, 1.00 mol amt.) in THF (70 mL) was added LiTMP in THF (0.50 M, 34.0 mL, 17.0 mmol, 2.4 mol amt.) at –78 °C and kept stirring at –50 °C for 5 h. After the reaction mixture was cooled to –78 °C, 1,1,2,2-tetrachloro-1,2-dibromoethane (6.00 g, 18.4 mmol, 2.6 mol amt.) in THF (15.0 mL) at –78 °C was transferred via Teflon cannula to the reaction mixture. The resulting brown suspension was left to reach

room temperature. After stirring at room temperature for 15 h, the resulting brown suspension was quenched with methanol. After the resulting brown solid was filtered, the solid was washed with chloroform to afford the titled compound as a brown solid (3.47 g, 88% yield). m.p.: > 350 °C. $^{1}$H NMR (400 MHz, TCE-$d_2$): $\delta$ 7.99 (d, 2H, $J$ = 8.8 Hz, Ar$H$), 8.08 (s, 2H, β position of thiophene ring), 8.43 (d, 2H, $J$ = 9.2 Hz, Ar$H$), 8.72 (d, 2$H$, $J$ = 9.2 Hz, Ar$H$), 8.82 (d, 2H, $J$ = 8.8 Hz, Ar$H$). $^{13}$C NMR could not be recorded due to the poor solubility. TOF HRMS (APCI+): Calcd for $C_{22}H_{10}Br_2S_2$ [M+H] 496.8669. Found, 496.8649. Anal. Calcd for $C_{22}H_{10}Br_2S_2$: C 53.03; H 2.02. Found: C 53.10; H 2.30.

**2,9-Didecylchryseno[2,1-*b*:8,7-*b*']dithiophene ($C_{10}$–ChDT)**

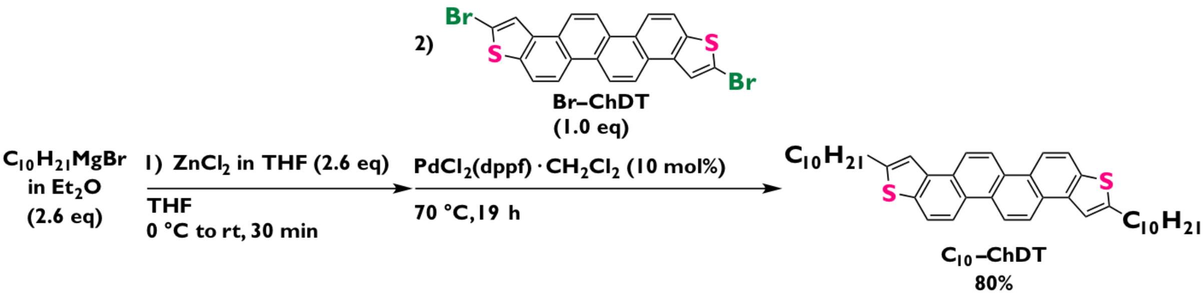


To a colorless solution of decylmagnesium bromide in diethylether (1.0 M, 1.55 mL, 1.55 mmol, 2.6 mol amt.) in THF (12.0 mL) was added $ZnCl_2$ in THF (1.0 M, 1.55 mL, 1.55 mmol, 2.6 mol amt.) at 0 °C. After stirring at room temperature for 30 min, **Br–ChDT** (295.7 mg, 0.593 mmol, 1.00 mol amt.) and $PdCl_2$(dppf)·$CH_2Cl_2$ (48.8 mg, 0.059 mmol, 10 mol%) were successively added at room temperature. The resulting brown suspension was warmed to 70 °C and stirred for 19 h. After cooling to room temperature, the resulting brown suspension was quenched with methanol and the solid was collected by filtration. The obtained brown solid was dissolved with *o*DCB, and then the solution was passed through a short pad of silica gel, followed by removal of organic solvent *in vacuo* to afford the titled compound as a white solid (293.5 mg, 80% yield). m.p.: 315–317 °C. $^{1}$H NMR (400 MHz, TCE-$d_2$): $\delta$ 0.90 (t, 6H, $J$ = 6.0 Hz, C$H_3$), 1.20–1.60 (m, 28H, C$H_2$), 1.79–1.92 (m, 4H, Ar-$CH_2$-C$H_2$), 3.06 (t, 4H, $J$ = 7.6 Hz, Ar-C$H_2$), 7.76 (s, 2H, β position of thiophene ring), 8.02 (d, 2H, $J$ = 9.2 Hz, Ar$H$), 8.47 (d, 2H, $J$ = 9.2 Hz, Ar$H$), 8.64 (d, 2H, $J$ = 9.2 Hz, Ar$H$), 8.84 (d, 2H, $J$ = 8.8 Hz, Ar$H$). $^{13}$C NMR (TCE-$d_2$): $\delta$ 14.0, 22.6, 29.2, 29.2, 29.4, 29.5, 29.6, 31.1, 31.4, 31.9, 118.9, 119.0, 121.1, 121.9, 123.3, 127.2, 127.8, 128.3, 137.2, 137.3, 147.7. TOF HRMS (APCI+): Calcd for

$C_{42}H_{53}S_2$ [M+H] 621.3589. Found, 621.3598. Anal. Calcd for $C_{42}H_{52}S_2$: C 80.23; H 8.44. Found: C 80.12; H 8.27.

**2,9-Bis(4-decylthiophen-2-yl)chryseno[2,1-*b*:8,7-*b*']dithiophene ($C_{10}$–Th–ChDT)**

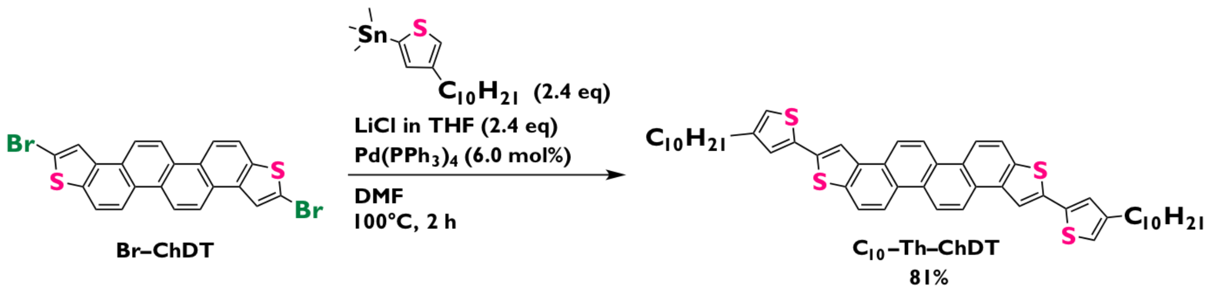


To brown suspension of **Br–ChDT** (402.0 mg, 0.807 mmol, 1.00 mol amt.) in DMF (16.0 mL) was added LiCl in THF (0.5 M, 4.8 mL, 2.4 mmol, 2.97 mol amt.), (4-decylthiophen-2-yl)trimethylstannane (787.0 mg, 2.02 mmol, 2.50 mol amt.), $Pd(PPh_3)_4$ (56.3 mg, 0.049 mmol, 6.0 mol%). The resulting brown suspension was heated to 100 °C and stirred at that temperature for 2 h. After the reaction mixture was cooled to room temperature, it was quenched with aqueous solution of potassium fluoride. The resulting suspension was filtered and the solid was washed with water and MeOH. The solid was dissolved with *o*DCB, passed through a short pad of silica gel and dried *in vacuo* to afford the titled compound as a white solid (510.8 mg, 81% yield). m.p.: 307–309 °C. $^1$H NMR (400 MHz, TCE-$d_2$): $\delta$ 0.88 (t, 6H, $J$ = 6.6 Hz, C$H_3$), 1.20-1.50 (m, 28H, C$H_2$), 1.67 (quin, 4H, $J$ = 7.4 Hz, Ar-CH$_2$-C$H_2$), 2.64 (t, 4H, $J$ = 7.4 Hz, Ar-C$H_2$), 6.94 (s, 2H, Ar*H* of thienyl group), 7.23 (s, 2H, Ar*H* of thienyl group), 8.04 (d, 2H, $J$ = 9.2 Hz, Ar*H*), 8.10 (s, 2H, Ar*H*), 8.52 (d, 2H, $J$ = 9.2 Hz, Ar*H*), 8.70 (d, 2H, $J$ = 9.2 Hz, Ar*H*), 8.86 (d, 2H, $J$ = 9.2 Hz, Ar*H*). $^{13}$C NMR (TCE-$d_2$): $\delta$ 14.0, 22.7, 29.2, 29.3, 29.4, 29.5, 29.6, 30.3, 31.5, 31.9, 117.5, 120.0, 121.0, 122.1, 123.4, 125.1, 125.2, 127.3, 128.0, 128.5, 137.8, 137.1, 137.6, 138.6, 147.2. TOF HRMS (APCI+): Calcd for $C_{50}H_{57}S_4$ [M+H] 785.3343. Found, 785.3366. Anal. Calcd for $C_{50}H_{56}S_4$: C 76.48; H 7.19. Found: C 76.55; H 7.11.

## 2. Theoretical Predictions

### 2.1 HOMO Configuration of the Reported Promising Semiconducting π-Electronic Cores

The Kohn–Sham energy levels of all of compounds in this work were calculated at the B3LYP/6-311G(d) level of theory with the SPARTAN 14 package, Wavefunction Inc. The reported promising semiconducting π-electronic cores including pentacene, [1]benzothieno[3,2-*b*][1]benzothiophene (**BTBT**), dinaphtho[2,3-*b*:2',3'-*f*]thieno[3,2-*b*]thiophene (**DNTT**), dinaphtho[2,3-*b*:2',3'-*d*]thiophene (**DNT–V**), and dinaphtho[2,3-*d*:2',3'-*d*']benzo[1,2-*b*:4,5-*b*']dithiophene(**DNBDT–N**), have the same HOMO configuration exhibiting the nodal change of their HOMO alternating along the molecular longitudinal axis as well as along the transverse one, as shown in Figure S1.

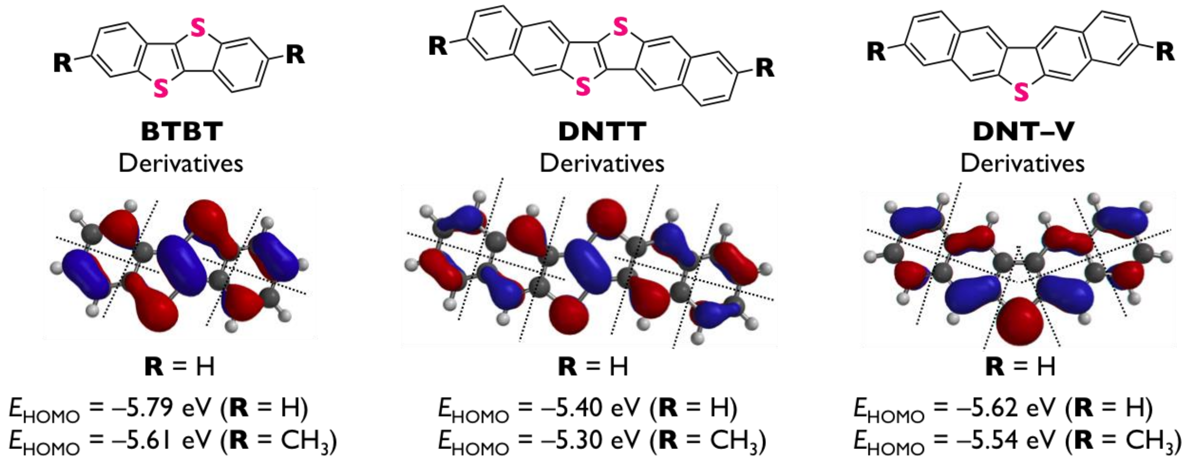


**Figure S1.** HOMO configurations and its energy levels of **BTBT**, **DNTT**, **DNT–V**, and their derivatives (calculated at the B3LYP/6-311Gd level).

## 2.2 HOMO Energy Levels of ChDT Derivatives

The HOMO level of **ChDT** core ($E_{HOMO}$ = –5.68 eV) is significantly higher than that of **DNT–W** core ($E_{HOMO}$ = –5.86 eV). The introduction of an electron-donating alkyl group into the **ChDT** core leads to an effective rise of the HOMO level (R = Me, $E_{HOMO}$ = –5.53 eV) because the HOMO coefficient of **ChDT** core at $\alpha$-position of terminal thiophene unit exists. Furthermore, the introduction of a thienyl group as a linker also lead a rise of HOMO level (R = Thienyl, $E_{HOMO}$ = –5.44 eV).

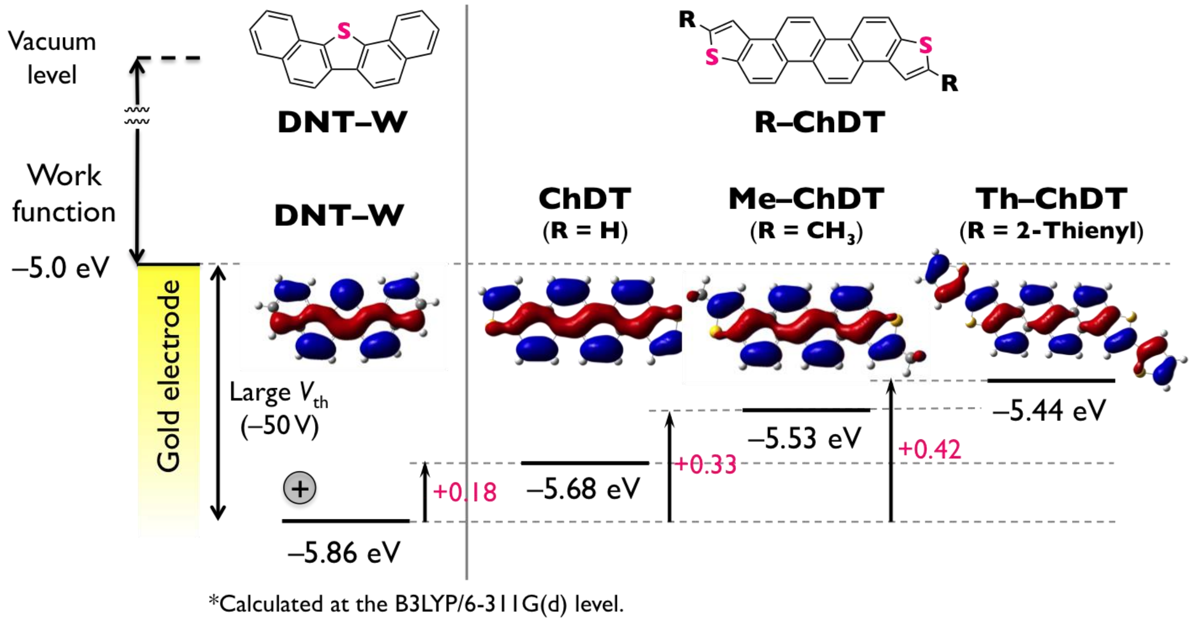


**Figure S2.** HOMO energy levels of **ChDT**, **Me–ChDT**, and **Th–ChDT**, together with **DNT–W** for comparison (calculated at the B3LYP/6-311G(d) level).

### 3. Solubility Test

To a weighed sample of around 1 mg was added 50 μL of toluene, repeatedly. The resulting suspension was shaken and sonicated at 60 °C. The total amount of solvent (mL) was converted into solubility in wt%. The results are summarized in Table S1.

**Table S1.** Solubility test of decyl-substituted **ChDT** derivatives in toluene at 60 °C, together with reported **$C_{10}$–DNTT** and **$C_{10}$–DNBDT–NW** for comparison.

| Compounds | Solubility in toluene (wt%) at 60 °C |
| --- | --- |
| **$C_{10}$–ChDT** | 0.068 |
| **$C_{10}$–Th–ChDT** | 0.13 |
| **$C_{10}$–DNTT** | 0.010 |
| **$C_{10}$–DNBDT–NW** | 0.033 |

**4. Ionization Potentials**

Photoelectron yield spectroscopy (PYS) was performed on a Sumitomo Heavy Industries Advanced Machinery PYS-202. For PYS measurement, thin films (ca. 100 nm) of all **ChDT** derivatives were thermally evaporated on ITO coated quartz substrates and measurements were performed in vacuum. The photoelectron yield spectra are depicted in Figure S3.

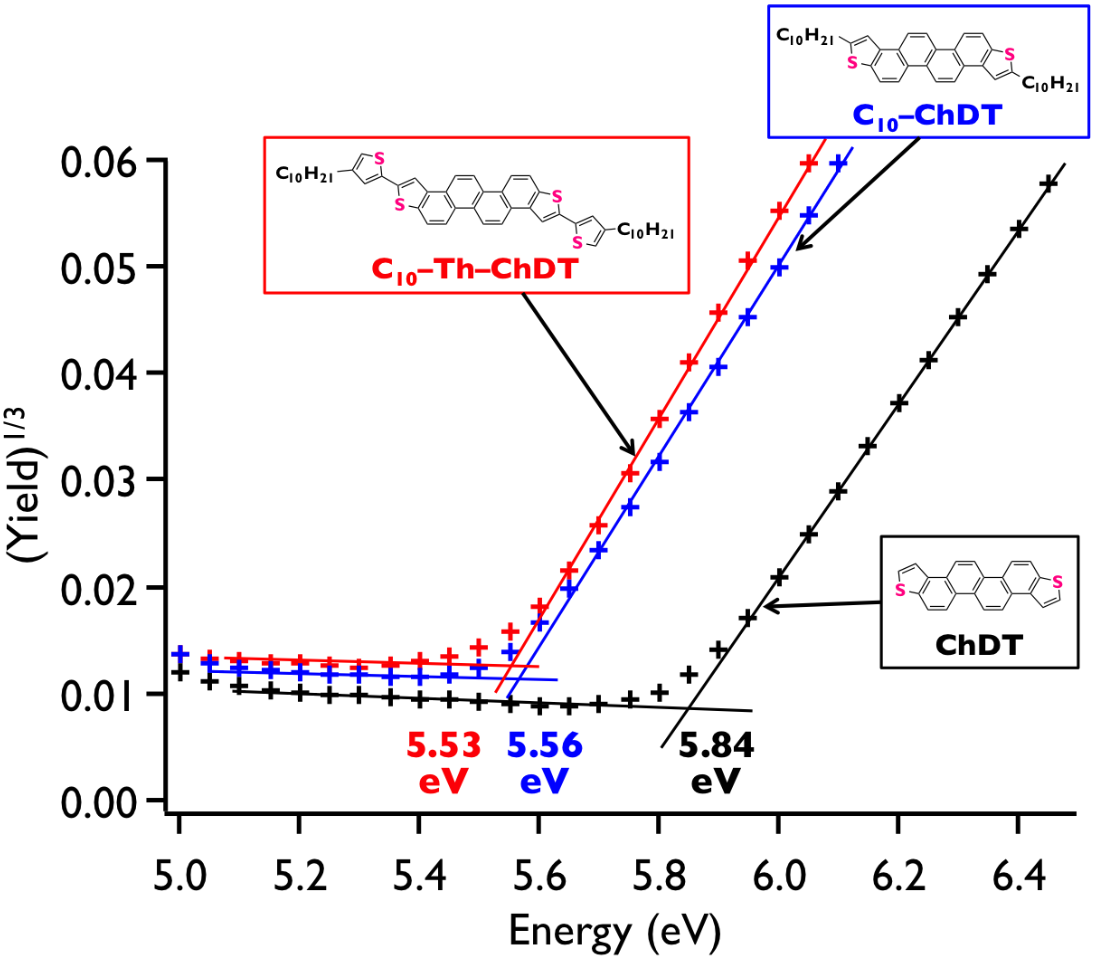


**Figure S3.** Photoelectron yield charts of **ChDT** derivatives in thin film after light illumination from a $D_2$ lamp *in vacuo*.

**5. Chemical Stability Test**

To evaluate the chemical stability of **ChDT** derivatives, time-dependent UV-vis absorption spectra were carried out for a period of 14 days. It was found that the spectra of π-extended derivative, **$C_{10}$–Th–ChDT** do not change over time, indicating that **ChDT** derivatives in this work have high chemical stability. Representative data for **$C_{10}$–Th–ChDT** are shown in Figure S4.

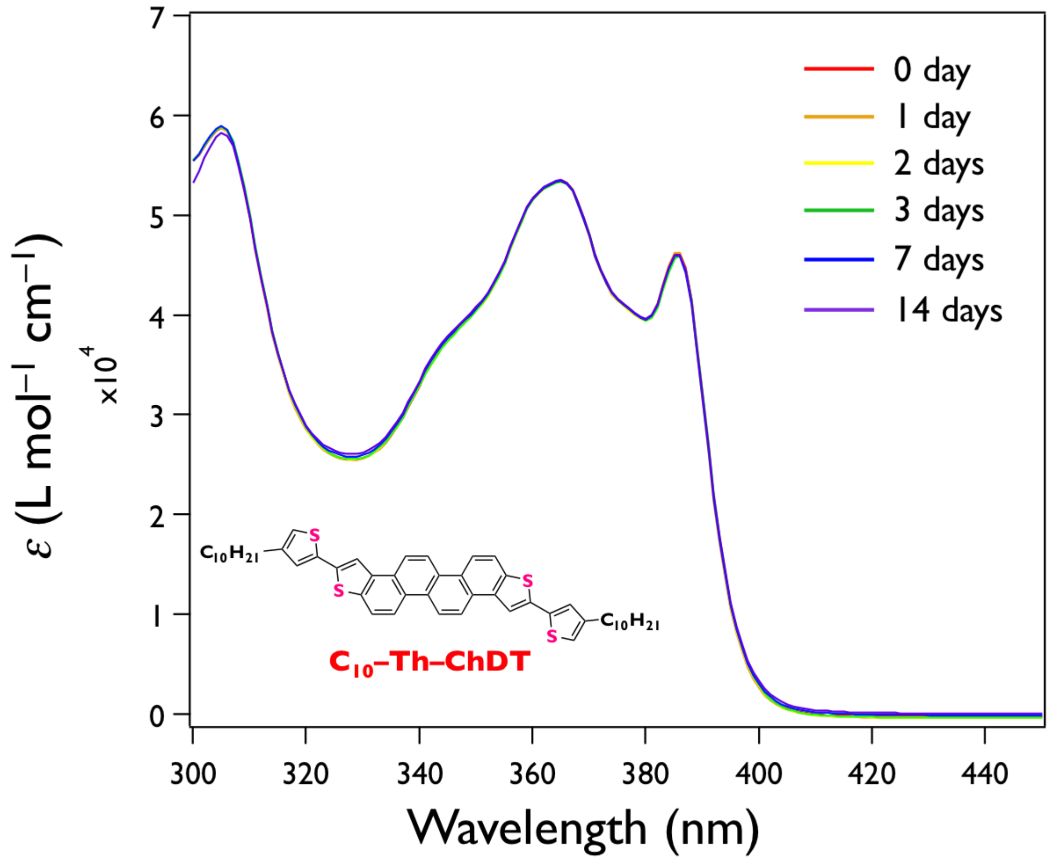


**Figure S4.** Time-dependent UV-vis absorption spectra of **$C_{10}$–Th–ChDT** in solution (1.1 x $10^{-5}$ M, 1,2-dichloroethane) for two weeks.

## 6. Thermal Analyses

### 6.1 Thermogravimetric Analysis (TGA)

TGA measurement was carried out with a Rigaku Thermo Plus EVO II TG 8121. Sample was placed in aluminum pan and heated at the rate of 5 ºC/min, under $N_2$ purge at a flow rate of 100 mL/min. $Al_2O_3$ was used as reference material. Prior to further purification to prepare device grade compounds, their thermal properties were investigated by TGA in the range of room temperature to 500 °C (See Figure S5). No thermal decomposition was observed in all of **ChDT** derivatives in that thermal range, implying that they can be purified by thermal sublimation.

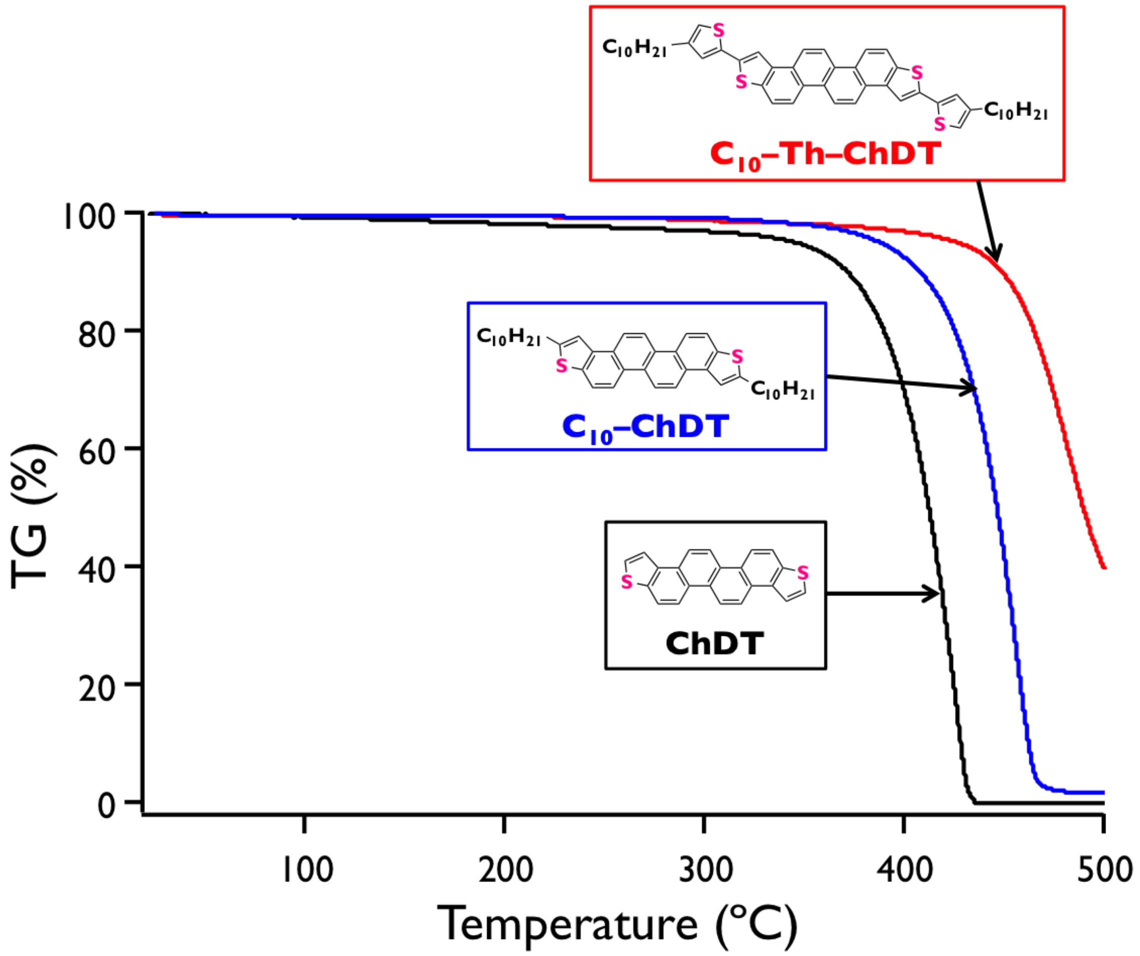


**Figure S5.** TGA plots of **ChDT** derivatives in the range of room temperature to 500 °C in a flow of nitrogen gas (scan rate: 5 °C/min, $N_2$ purge: 100 mL/min).

## 6.2 Differential Scanning Calorimetry (DSC)

DSC measurement was carried out with a Rigaku Thermo Plus EVO IIDSC 8231. Sample was placed in aluminum pan and heated at the rate of 5 ºC/min, under $N_2$ purge at a flow rate of 100 mL/min. $Al_2O_3$ was used as reference material. The DSC measurements could lead to phase-transition data for the endothermic process (either from solid to liquid or from solid to liquid crystal).

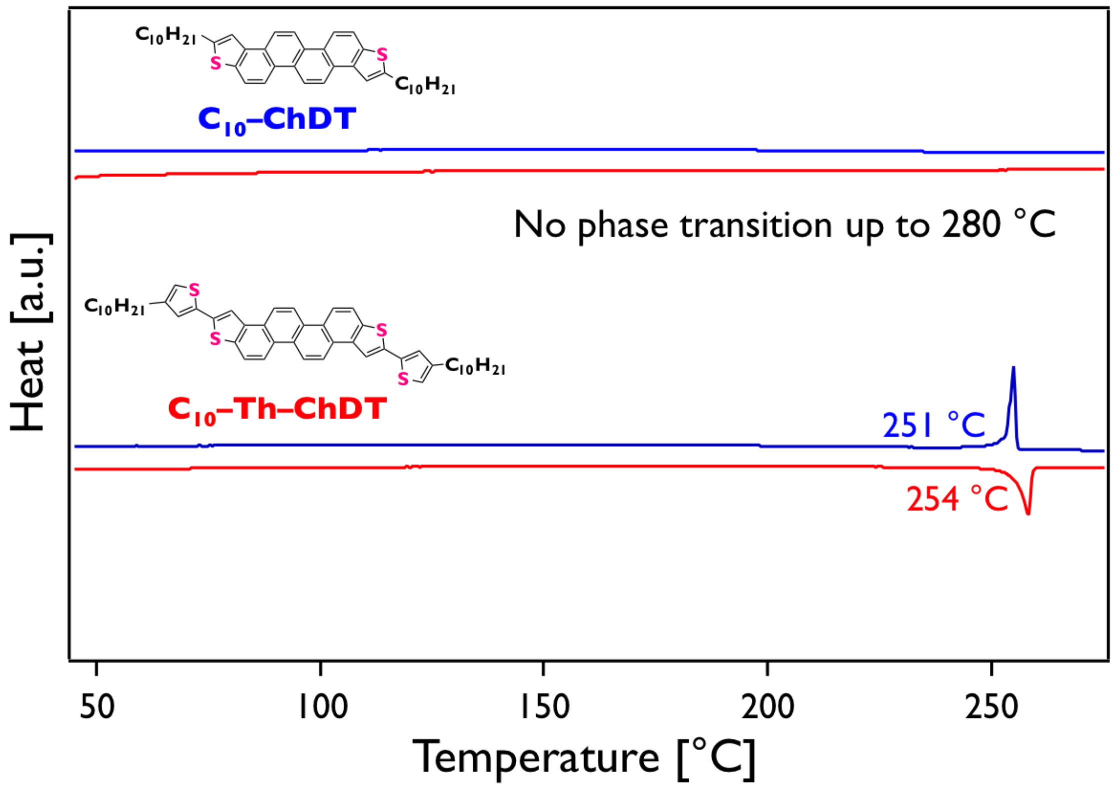


**Figure S6.** DSC plots of **ChDT** derivatives in the range of 40 to 280 °C in a flow of nitrogen gas (scan rate: 5 °C/min, $N_2$ purge: 100 mL/min).

## 7. Single-Crystal Analyses

It is crucially important to estimate the potential of the semiconductors by examining not only their molecular structure but also their packing structure in the solid state. Single crystals were obtained by means of either physical vapor transport (PVT) technique or recrystallization from certain organic solvents. That of **ChDT** was grown by PVT (315 °C and 220 °C in argon flow of 40 ccm). Those of **$C_{10}$–ChDT** and **$C_{10}$–Th–ChDT** were grown by gradual diffusion of isopropanol into toluene solution. Single-crystal diffraction data were collected on a Rigaku R-AXIS RAPID II imaging plate diffractometer with Cu$K\alpha$ radiation for **ChDT**, **$C_{10}$–ChDT** and **$C_{10}$–Th–ChDT**. The molecular structures (ellipsoid type) in the front and side views, the intermolecular interactions and short contacts, and packing structures of **ChDT** and **$C_{10}$–ChDT** are shown in Figure S7–12.

**Table S2.** Crystal data for **ChDT**, **$C_{10}$–ChDT**, and **$C_{10}$–Th–ChDT**.

| | **ChDT** | **$C_{10}$–ChDT** | **$C_{10}$–Th–ChDT** |
|---|---|---|---|
| formula | $C_{22}H_{12}$ $S_2$ | $C_{42}H_{52}S_2$ | $C_{50}H_{56}S_4$ |
| FW | 340.46 | 620.99 | 785.23 |
| T/K | 296 | 243 | 243 |
| wavelength/Å | 1.54187(Cu$K\alpha$) | 1.54187(Cu$K\alpha$) | 1.54187(Cu$K\alpha$) |
| color | colorless | colorless | colorless |
| crystal size, mm | 0.541x0.344x0.001 | 0.500x0.400x0.001 | 0.150x0.150x0.001 |
| crystal system | monoclinic | monoclinic | monoclinic |
| space group | $P2_1/c$ | C2/$c$ | $P2_1/c$ |
| $a$/Å | 15.0548(14) | 67.06(3) | 41.485(4) |
| $b$/Å | 5.9744(6) | 4.8610(18) | 7.3716(8) |
| $c$/Å | 8.5346(8) | 10.719(4) | 6.9093(7) |
| $\alpha$/deg | 90 | 90 | 90 |
| $\beta$/deg | 101.176(7) | 90.302(10) | 93.852(7) |
| $\gamma$/deg | 90 | 90 | 90 |
| $V$/Å$^3$ | 753.07(12) | 3494(2) | 2108.2(4) |
| $Z$ | 2 | 4 | 2 |
| $D_X$/g cm$^{-3}$ | 1.501 | 1.180 | 1.237 |
| $\mu$/mm$^{-1}$ | 3.166 | 1.574 | 2.315 |
| reflections collected | 7365 | 9197 | 23479 |
| unique reflections | 1383 | 1266 | 3855 |
| refined parameters | 109 | 200 | 245 |
| GOF on $F^2$ | 1.001 | 1.127 | 0.846 |
| R1 [I > 2$\sigma(I)$][a] | 0.0812 | 0.1384 | 0.0756 |
| w$R$2 (all data)[b] | 0.2290 | 0.3731 | 0.1891 |
| $\Delta\rho_{min, max}$ / e Å$^{-3}$ | -0.29, 0.64 | -0.23, 0.36 | -0.23, 0.23 |
| CCDC number | 1544287 | 1544288 | 1544289 |

[a] $R1 = \Sigma ||Fo|–|Fc|| / \Sigma |Fo|$, [b] $wR2 = [\Sigma (w(Fo^2–Fc^2)^2/\Sigma w(Fo^2)^2)^{1/2}$

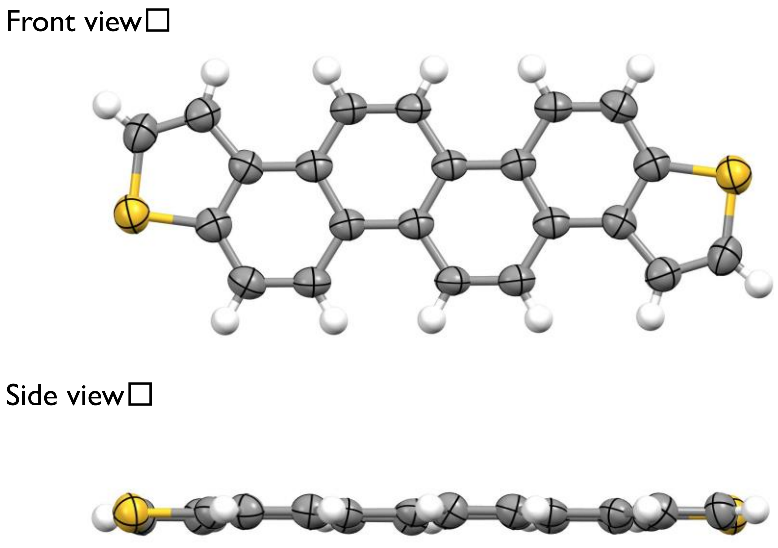


**Figure S7.** Molecular structures (ellipsoid style) of **ChDT**.

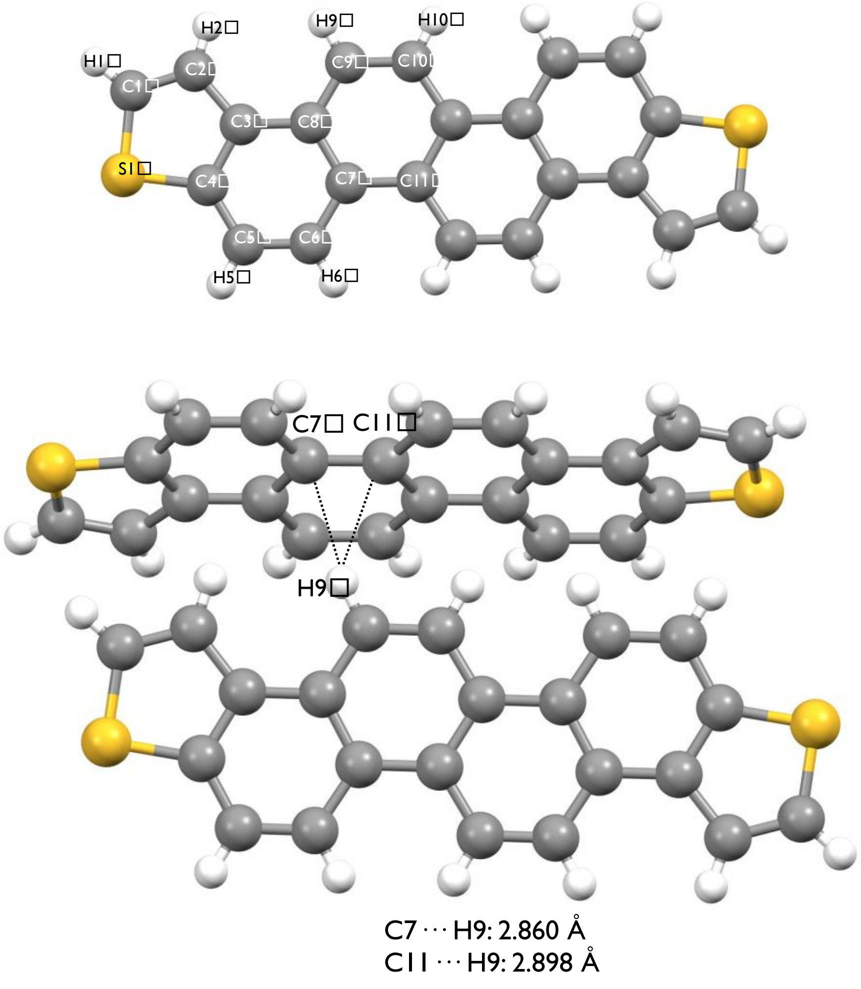


**Figure S8.** Intermolecular interactions and short contacts of **ChDT**.

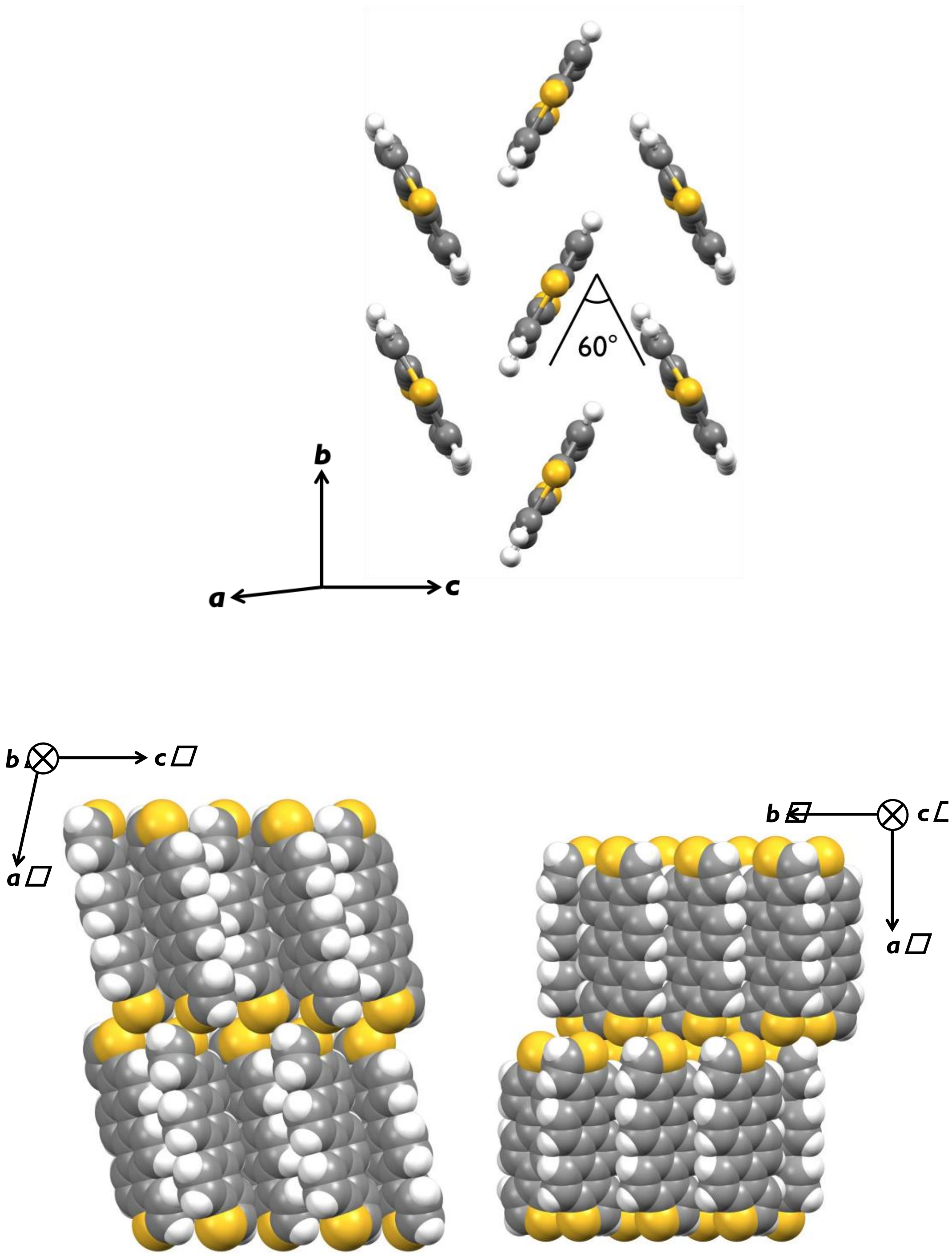


**Figure S9.** Packing structures of **ChDT**.

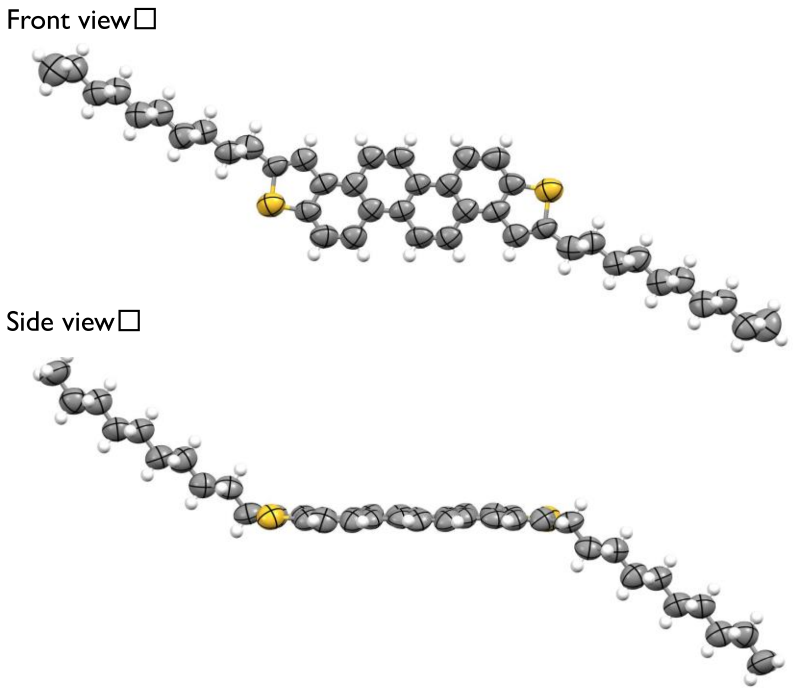


**Figure S10.** Molecular structures (ellipsoid style) of **$C_{10}$–ChDT**.

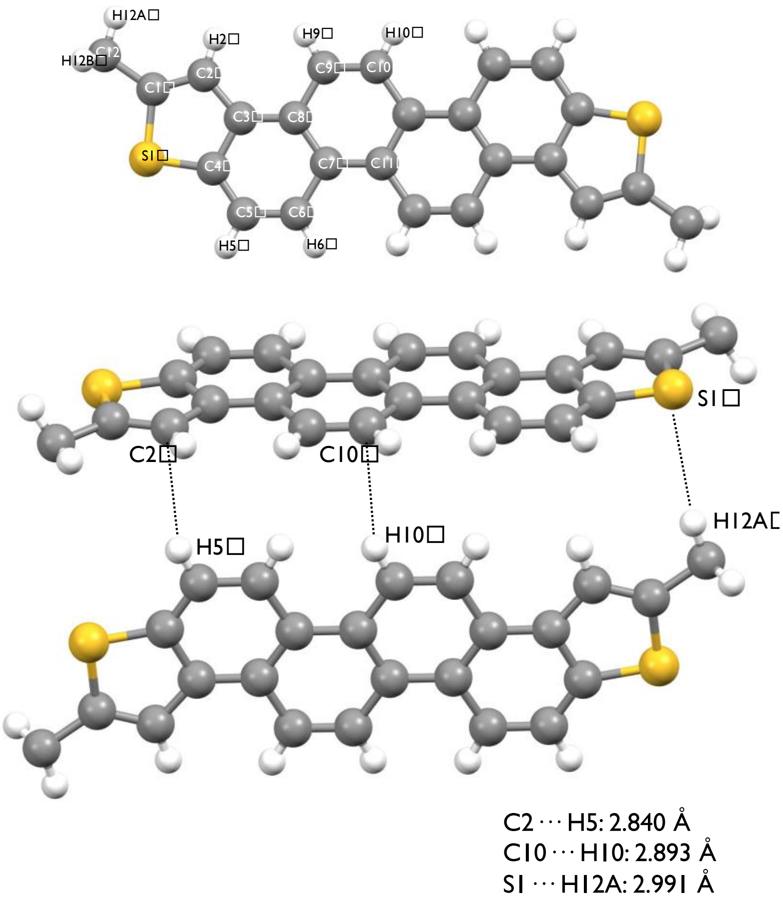


**Figure S11.** Intermolecular interactions and short contacts of **$C_{10}$–ChDT**.

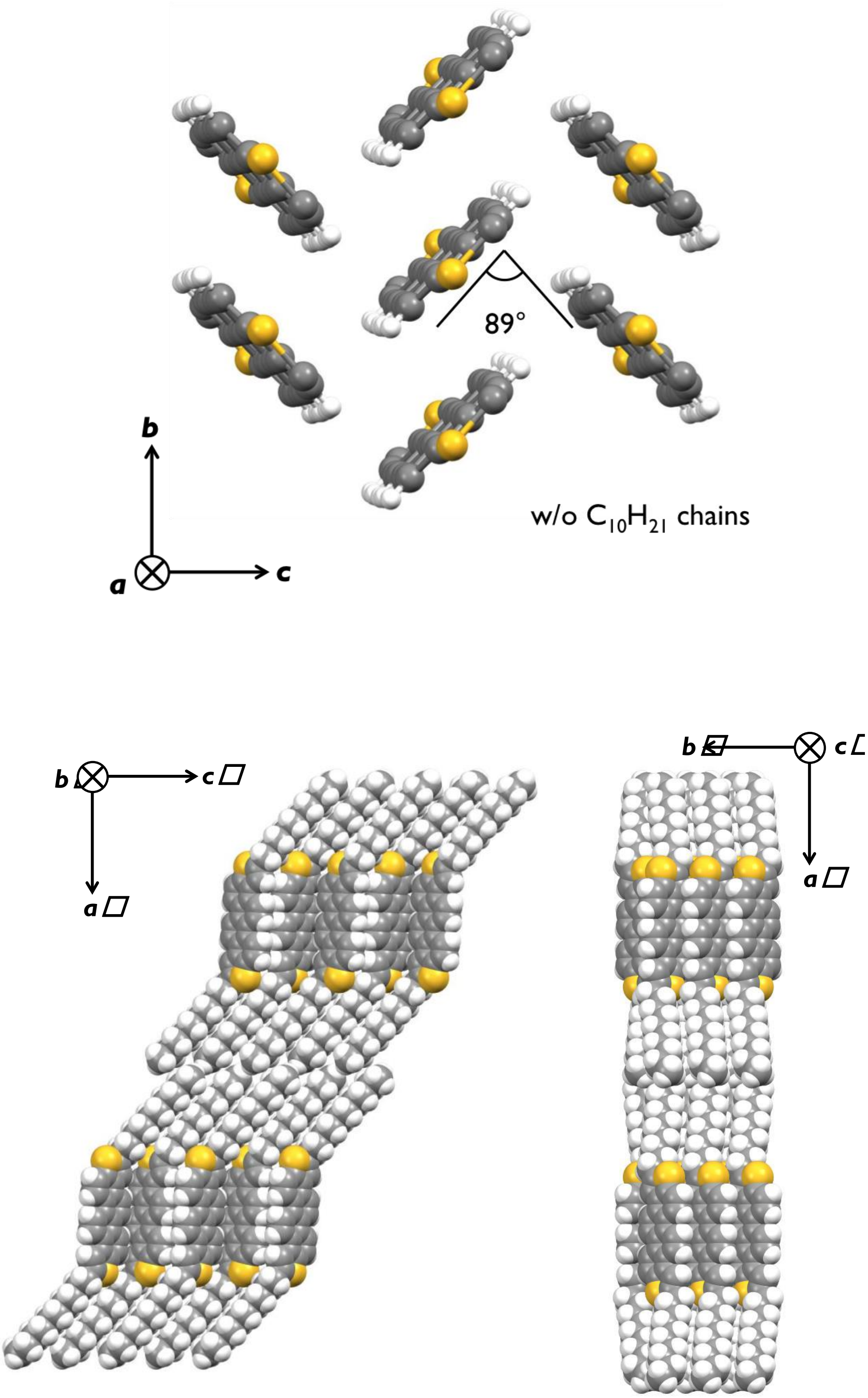


**Figure S12.** Packing structures of **$C_{10}$–ChDT**.

## 8. Transfer Integral and Band Calculations

Based on their packing structures, the transfer integral (*t*) values of the HOMOs between neighboring molecules were estimated by density functional theory at the PBEPBE/6-31G(d) level, as illustrated in Figure S13. Figure S14 shows transfer integral of **$C_{10}$–Th–ChDT** packing structure depending on the displacement from its original packing structures in column direction, indicating that **$C_{10}$–Th–ChDT** is less susceptible to the displacement parameter in the direction of molecular longitudinal axis.

To further understand the carrier transporting capabilities in the bulk state, their electronic band structure were also calculated at the same level as the intermolecular electronic couplings using the periodic boundary condition at the PBEPBE/6-31G(d) level. Electronic band structure calculations were conducted based on the packing structure by way of 2-dimensional periodic boundary condition (Figure S15–17). The energies are plotted and labeled as $\Gamma = (0, 0, 0)$, $S = (0.5, 0.5, 0)$, $T = (0, 0.5, 0.5)$, $U = (0.5, 0, 0.5)$, $X = (0.5, 0, 0)$, $Y = (0, 0.5, 0)$, $Z = (0, 0, 0.5)$ in the crystallographic coordinates. Effective hole masses were calculated as $m^* = \hbar^2 \left(\frac{\partial^2 E(k)}{\partial k^2}\right)^{-1}$ along respective directions (Figure S13).

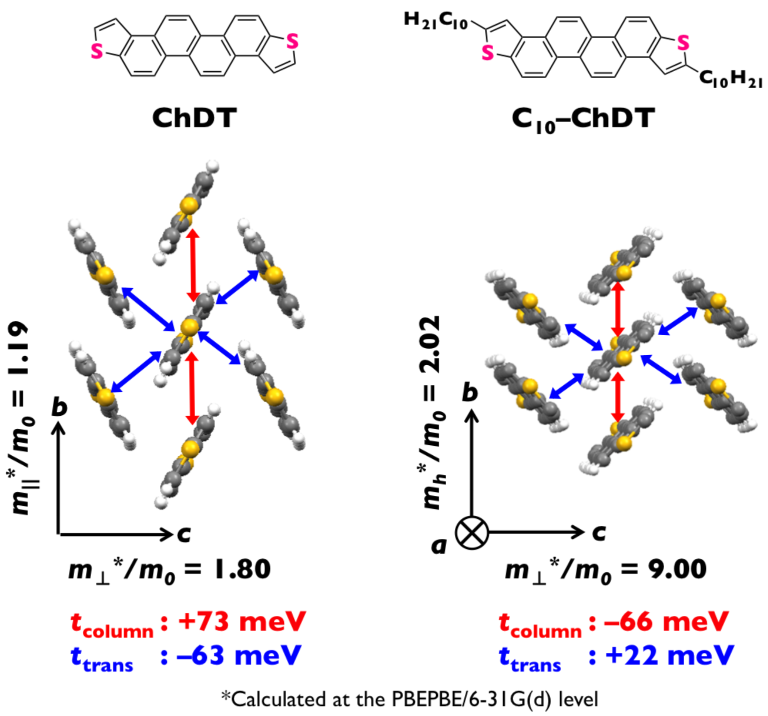


**Figure S13.** Transfer integrals and effective masses of **ChDT** and **$C_{10}$–ChDT**.

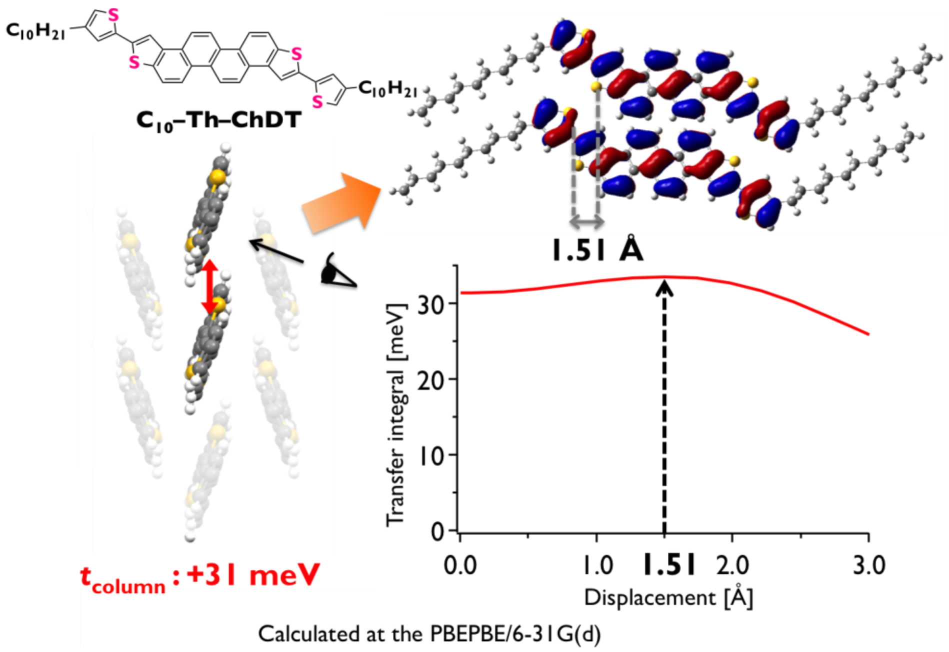


**Figure S14.** Transfer integral of **$C_{10}$–Th–ChDT** packing structure depending on the displacement from its original packing structures in column direction.

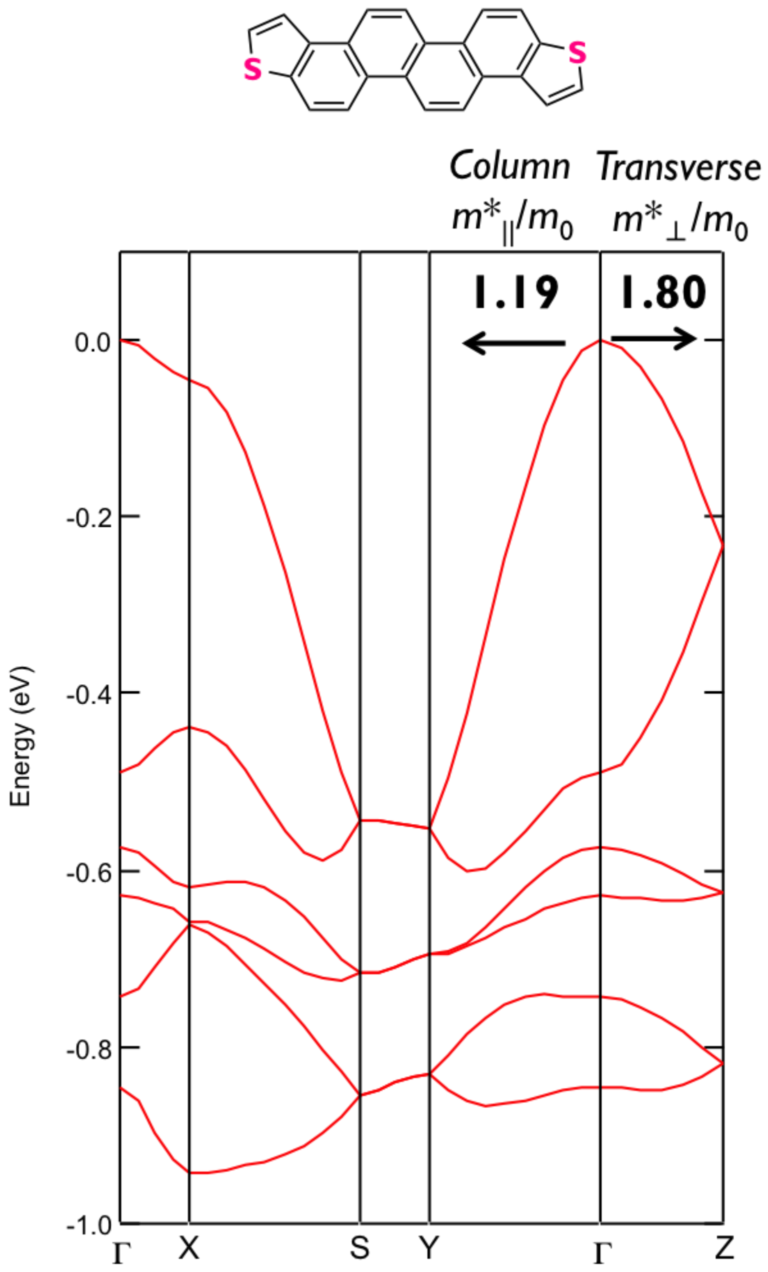


**Figure S15.** Electronic band structure of **ChDT** crystal and calculated effective mass ($m^*/m_0$). The top of HOMO band is set to 0 eV.

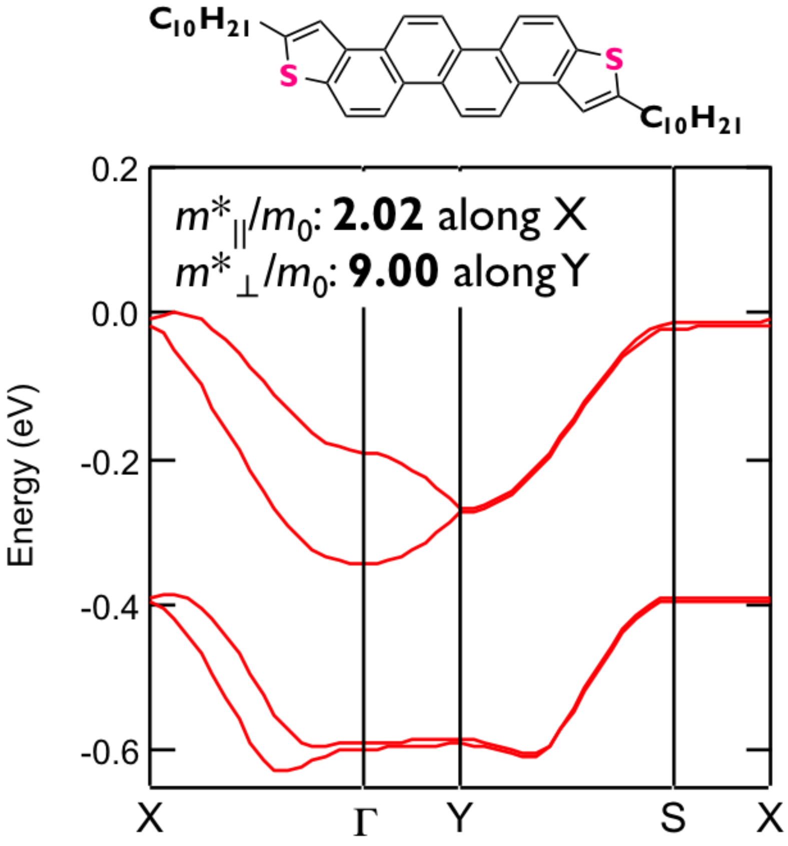


**Figure S16.** Electronic band structure of **$C_{10}$–ChDT** crystal and calculated effective mass ($m^*/m_0$). The top of HOMO band is set to 0 eV.

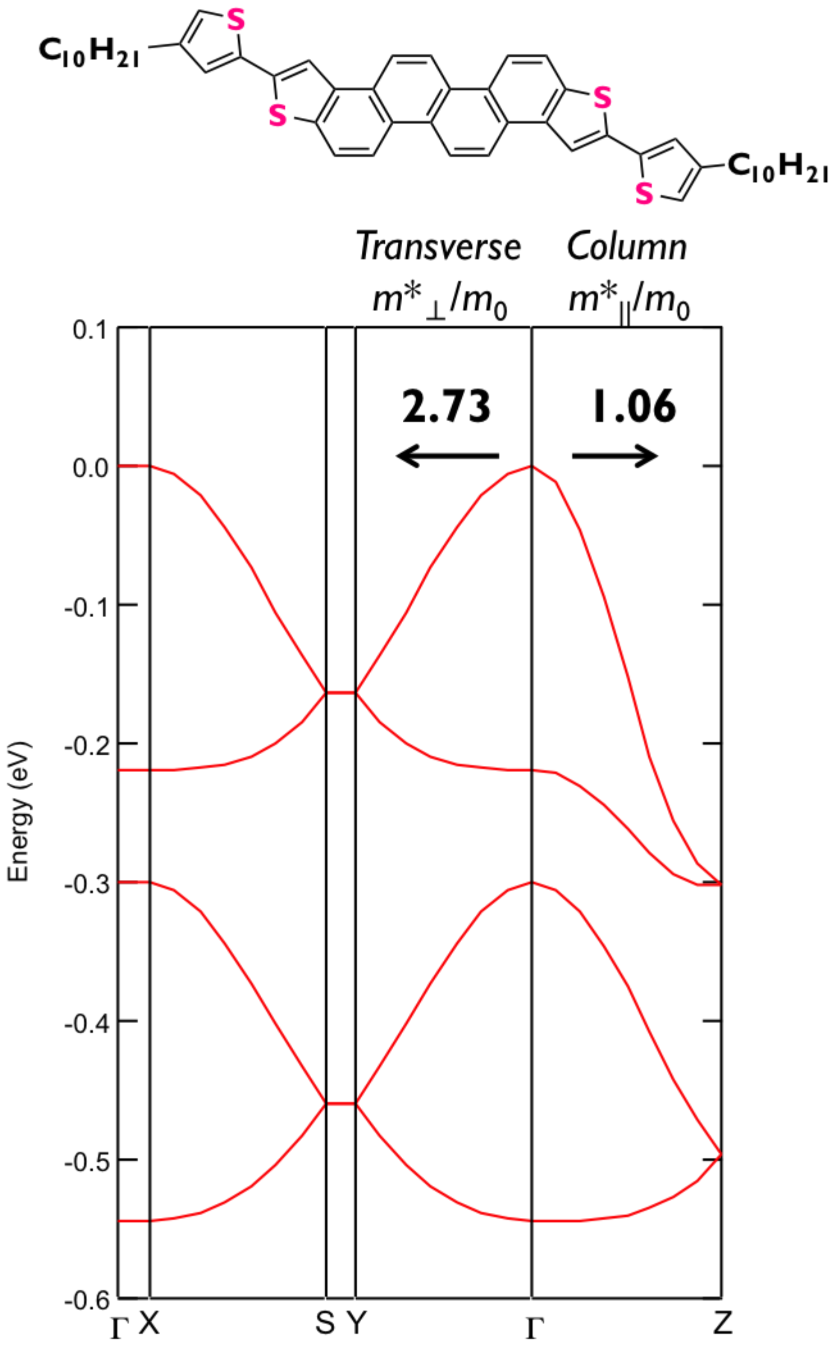


**Figure S17.** Electronic band structure of **$C_{10}$–Th–ChDT** crystal and calculated effective mass ($m^*/m_0$). The top of HOMO band is set to 0 eV.

## 9. Calculations for Amplitude of Translational Motions

The amplitude of translational motion of a molecule in the aggregated structure can be estimated as follows. First, the total energy of the cluster as shown in Figure S18a) for the experimental structure is calculated using the density functional theory at the B3LYP/6-31G(d) level with the van der Waals correction in the framework of DFT-D approach. Then, change in total energy is computed under a rigid molecule approximation as a function of displacement of the molecule surrounded by the black square in Figure S18a). The displacements along the column, transverse, and out-of-plane directions are investigated here. As shown in Figure S18b), the amplitude of translational motion at the temperature $T$ = 300K is obtained as the displacement where the total energy is increased up to $k_B T$ = 25.7 meV. The calculated amplitudes are summarized in Table S3.

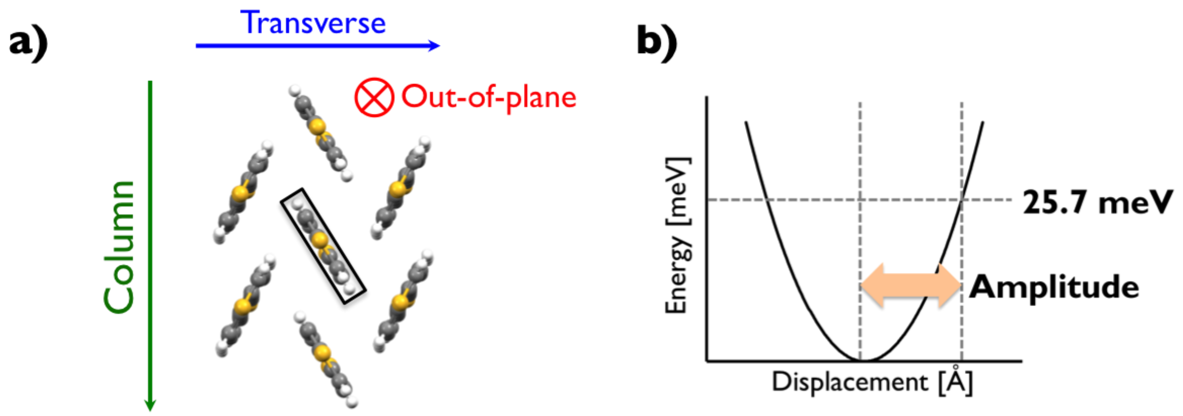


**Figure S18**. a) Schematic picture of displacement along columnar, transverse, and long-axis directions in the herringbone structure. b) The total energy calculated for the cluster shown in a) as a function of the displacement, using the density functional theory at the B3LYP/6-31G(d) level with the van der Waals correction in the framework of DFT-D approach.

**Table S3**. Estimated amplitudes of displacement along columnar, transverse and long-axis directions for **ChDT**, **DNTT**, and pentacene.

| Material | Amplitude of displacement (Å) | | |
|---|---|---|---|
| | Column | Transverse | Out-of-plane |
| **ChDT** | 0.120 | 0.093 | 0.296 |
| **DNTT** | 0.128 | 0.083 | 0.269 |
| **Pentacene** | 0.136 | 0.119 | 0.348 |

## 10. OFET Device Fabrication and Evaluation Procedure

Organic field-effect transistors based on organic semiconducting crystals were fabricated with the lamination method for **ChDT** and the edge-casting method for **$C_{10}$–ChDT** and **$C_{10}$–Th–ChDT**. The surfaces of a heavily-doped silicon wafers with thermally-oxidized $SiO_2$ (500 nm) were pretreated with self-assembled monolayers (SAMs) prior to each method; heptadecafluorodecyltrimethoxysilan (FDTS-SAM) for the lamination method and *β*-phenylethyltrimethoxylsilane (*β*-PTS-SAM) for the edge-casting method (Figure S19). The FDTS-SAM can avoid contamination adhesion onto the surface and positively shifting the threshold voltage ($V_{th}$) by its dipole moment. The *β*-PTS-SAM can improve wettability and resultant solution-processability. Onto the FDTS-SAM treated substrates, **ChDT** crystals, which were grown by the PVT technique, were softly laminated. On the other hand, on the *β*-PTS-SAM treated substrates, a droplet of solution of **$C_{10}$–ChDT** or **$C_{10}$–Th–ChDT** was put at the edge of a liquid-sustaining piece. The organic crystalline films grew on top of the solution surface, during the solvent evaporated from opposite side of the sustaining piece, to land softly on the substrate. After removing the sustaining piece, the film on the substrate was dried under heating in vacuum.

**$C_{10}$–ChDT** solution (0.05 wt%) in 2,5-dimethoxytoluene was dropped on the substrate at 100 ºC, and the film was dried at 100 ºC in vacuum for 12 hours. **$C_{10}$–Th–ChDT** solution (0.10 wt%) in 1-chloronaphthalene was dropped on the substrate at 70 ºC and the film was dried at 60 ºC in vacuum for 12 hours. Onto the both crystal-films, $F_4$-TCNQ (2 nm) and Au (50–80 nm) were thermally deposited through a shadow mask to construct bottom-gate-top-contact architecture. Finally, crystals were shaped into rectangular channels by laser etching to determine channel length and width for correct evaluation of mobility. In the device, $SiO_2$ layer acts as a gate insulator, doped silicon as a gate electrode, and Au as contact electrodes (source and drain electrode). The layer of $F_4$-TCNQ, electron-accepting material, was introduced between organic semiconductor and contacts to reduce parasitic contact resistance.

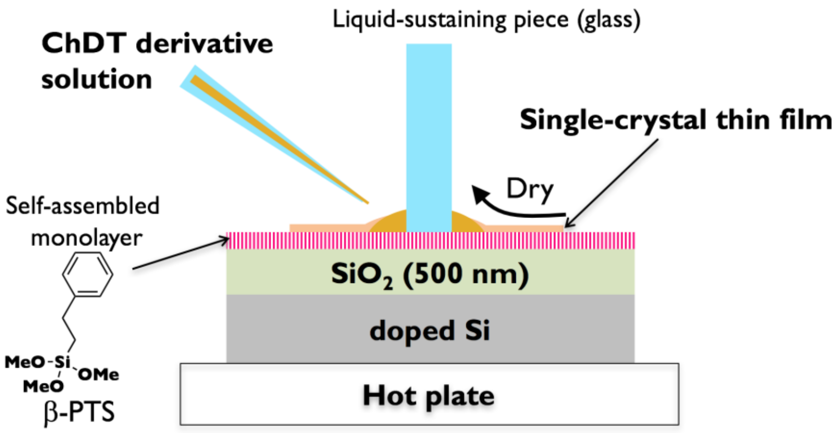


**Figure S19.** Illustration of the edge-casting method.

Transistor characterizations were carried out using Keithley 4200 semiconductor parameter analyzer. Transistor characteristics for **ChDT** and **$C_{10}$–ChDT** are shown in Figure S20 and Figure S21, respectively. After checking small hysteresis in transfer curve and saturation of drain current in output characteristics, field-effect mobilities ($\mu_{FET}$) in the saturation regime were evaluated using the following the equation:

$$I_D = (WC_i/2L)\ \mu_{FET}\ (V_G - V_{th})^2$$

Where $C_i$ is the capacitance of gate insulator and $V_{th}$ is the threshold voltage. The highest mobility values are summarized in main text.

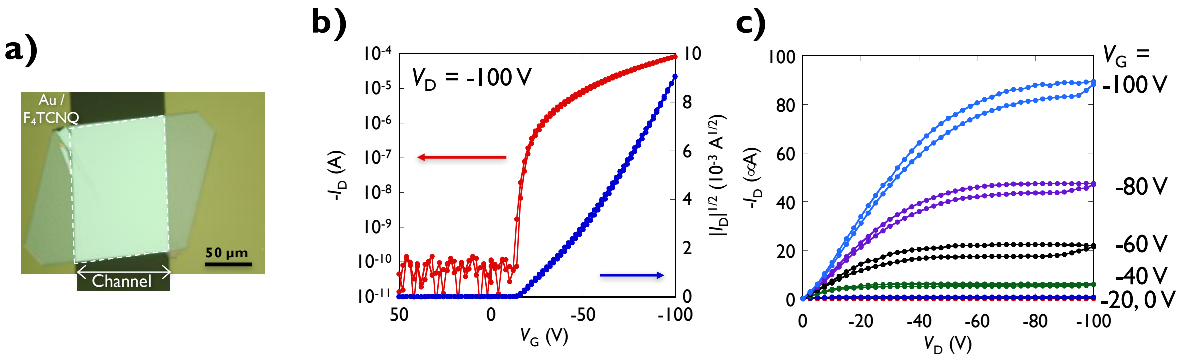


**Figure S20.** Typical **ChDT** single-crystal transistor. a) Polarized microscopic image of the device, b) transfer characteristics in saturation region, and c) output characteristics.

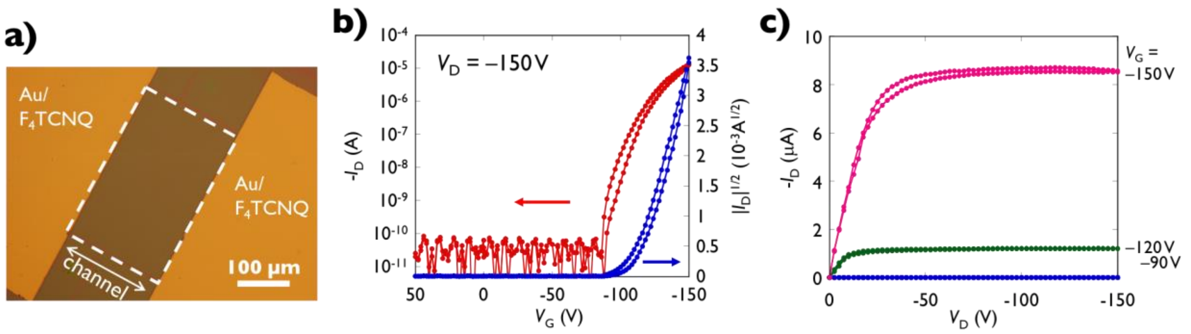


**Figure S21.** Typical **$C_{10}$–ChDT** single-crystal transistor. a) Microscopic image of the device, b) transfer characteristics in saturation region, and c) output characteristics.

## 11. Atomic Force Microscopy

To reveal the surface morphorogy of solution-processed single-crystalline film for **C$_{10}$–ChDT** and **C$_{10}$–Th–ChDT**, atomic force microscopy (AFM) were carried out with Shimadzu SPM-9700HT. Since the device channels are formed as mono-domain signle crystal film, crystal steps were observed around channels. The AFM images are shown in Figure S22 for **C$_{10}$–ChDT** and Figure S23 for **C$_{10}$–Th–ChDT** crystalline films. The films have molecularly-flat terraces with one- or two-molecular steps. The heights correspond to each single-crystal structural data (Table S2), which is consistent with XRD experiments (Figure S24). Moreover, for **C$_{10}$–ChDT** crystal, some cores can be clearly observed on the terrace.

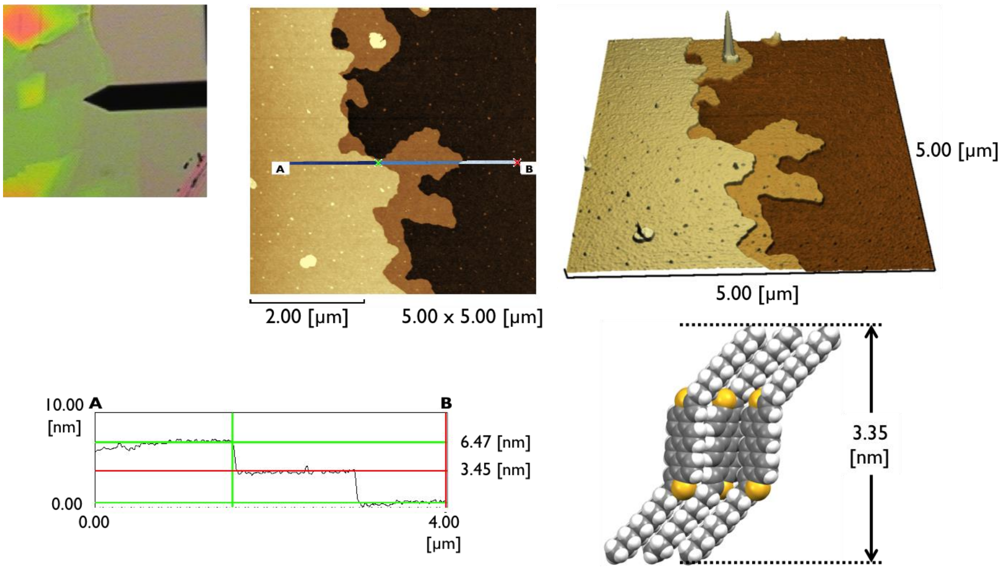


**Figure S22.** AFM images of **C$_{10}$–ChDT** solution-processed crystalline film.

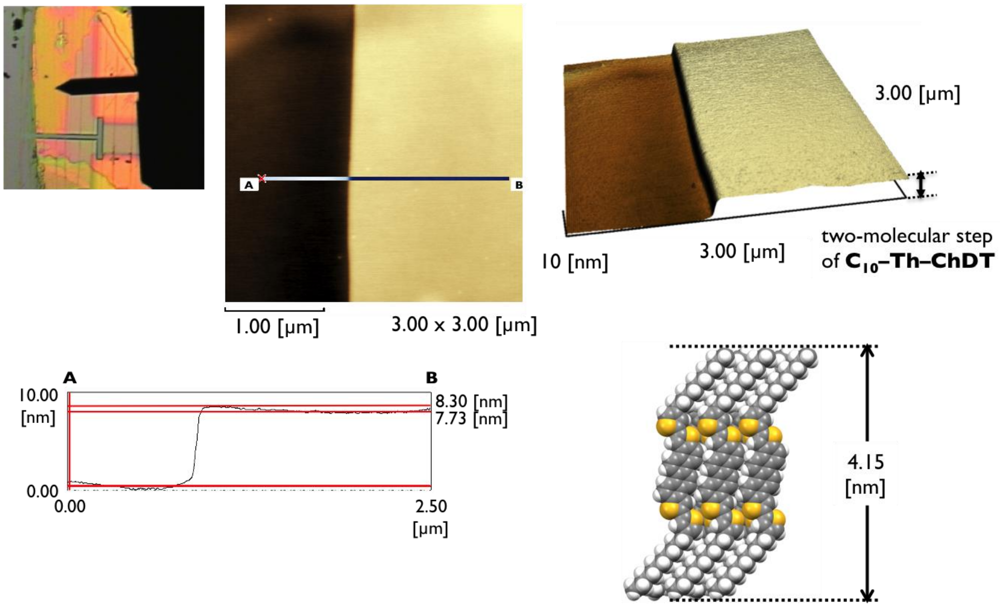


**Figure S23.** AFM images of **C$_{10}$–Th–ChDT** solution-processed crystalline film.

**12. X-ray-diffraction Measurements for Solution-crystallized Thin Film**

In order to determine the crystal directions in the actual devices, X-ray-diffraction measurements were carried out with transmission X-ray. Diffraction data collected on an imaging plate are shown in Figure S24 for **$C_{10}$–Th–ChDT**. Under the assumption that the crystal structure of the crystalline films was the same as the structures unveiled by the single-crystal structure analysis, the Laue spots would be assigned as described in the figures. In the **$C_{10}$–Th–ChDT** device, the *a* axis is almost perpendicular to and the *bc* place is parallel to the substrate surface. The direction of the crystal growth and the channel direction are almost the *c* axis, which means carriers transport along the direction of smaller effective mass ($m^*_{\parallel}/m_0$ = 1.06).

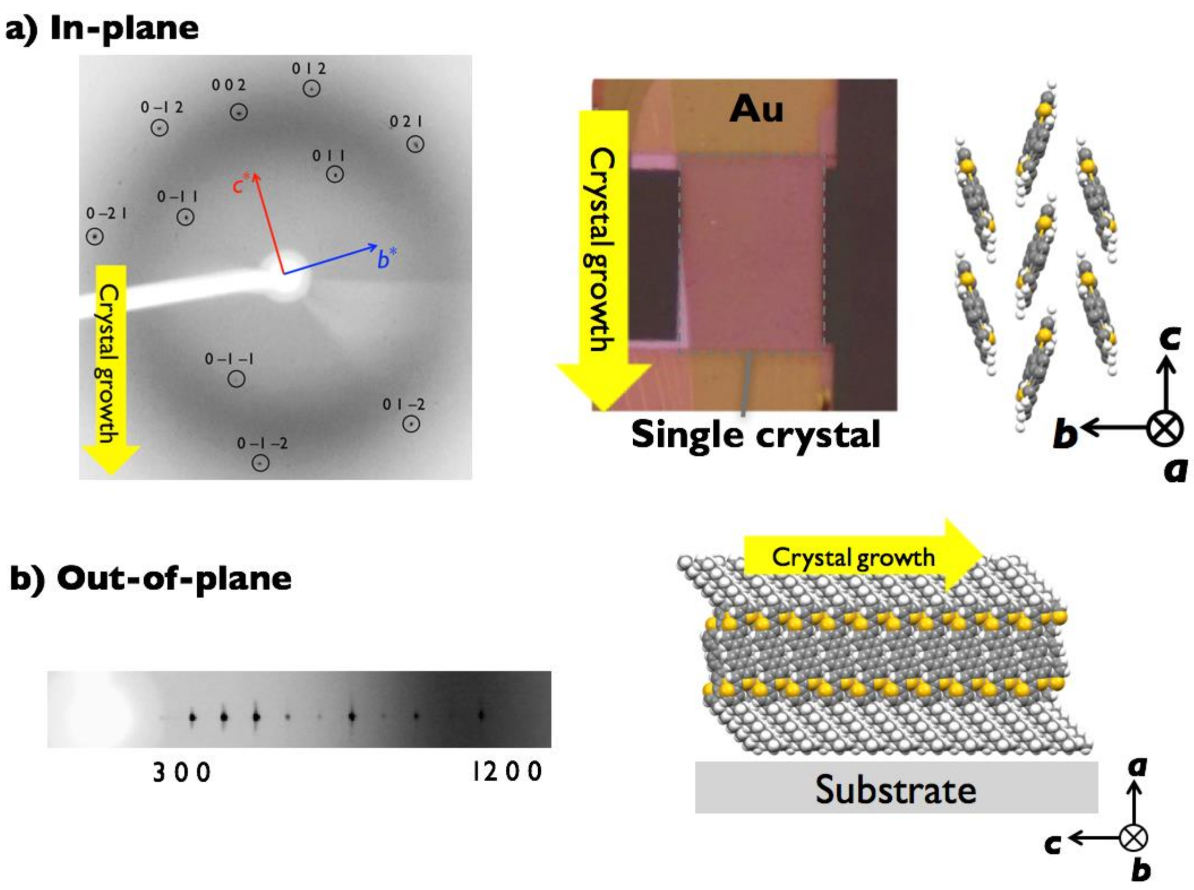


**Figure S24.** a) In-plane and b) out-of-plane Laue spots on an imaging plate for edge-casted **$C_{10}$–Th–ChDT** crystalline film with crystal structures.

## 13. References

[S1] S. Shinamura, I. Osaka, E. Miyazaki, A. Nakao, M. Yamagishi, J. Takeya, K. Takimiya, *J. Am. Chem. Soc.* **2011**, *133*, 5024.
